# The impact of spike timing precision and spike emission reliability on decoding accuracy

**DOI:** 10.1038/s41598-024-58524-7

**Published:** 2024-05-08

**Authors:** Wilten Nicola, Thomas Robert Newton, Claudia Clopath

**Affiliations:** 1 University of Calgary, Calgary, Canada; 2 Department of Cell Biology and Anatomy, Calgary, Canada; 3 Hotchkiss Brain Institute, Calgary, Canada; 4 Department of Bioengineering, Imperial College London, London, UK

**Keywords:** Computational neuroscience, Mathematics and computing, Applied mathematics, Dynamical systems

## Abstract

Precisely timed and reliably emitted spikes are hypothesized to serve multiple functions, including improving the accuracy and reproducibility of encoding stimuli, memories, or behaviours across trials. When these spikes occur as a repeating sequence, they can be used to encode and decode a potential time series. Here, we show both analytically and in simulations that the error incurred in approximating a time series with precisely timed and reliably emitted spikes decreases linearly with the number of neurons or spikes used in the decoding. This was verified numerically with synthetically generated patterns of spikes. Further, we found that if spikes were imprecise in their timing, or unreliable in their emission, the error incurred in decoding with these spikes would be sub-linear. However, if the spike precision or spike reliability increased with network size, the error incurred in decoding a time-series with sequences of spikes would maintain a linear decrease with network size. The spike precision had to increase linearly with network size, while the probability of spike failure had to decrease with the square-root of the network size. Finally, we identified a candidate circuit to test this scaling relationship: the repeating sequences of spikes with sub-millisecond precision in area HVC (proper name) of the zebra finch. This scaling relationship can be tested using both neural data and song-spectrogram-based recordings while taking advantage of the natural fluctuation in HVC network size due to neurogenesis.

## Introduction

The firing of spikes is metabolically costly, with the brain consuming approximately 20% of the body’s energy at any time^1^. Indeed, studies show that the firing and transmission of action potentials via synapses consumes the majority of the predicted energy used by cells in the cerebral cortex^2^. Thus, to maximize the use of any particular spike in encoding/decoding stimuli across trials, a neural circuit may devote resources to increasing the reliability of emitting existing spikes and the timing precision of these emitted spikes, rather than adding extra spikes. This may also be a useful strategy in improving the performance of neuromorphic circuits, which use hardware instantiated spiking neurons for computations^3,4^.

Indeed, there are existing examples in neuroscience where neural circuits prioritize the reliability of spike emission and the precision of spike times, rather than encoding with a large volume of spikes. For example, the neural circuits controlling the singing behaviour of the zebra finch prioritize spike timing precision and emission reliability. The zebra finch singing behaviour is dependent on multiple nuclei post-learning. Here, we focus on the HVC (proper name), the Robust Nucleus of the Archopallium (RA), and the hypoglossal nucleus^5–11^. During the approximately half second bout of singing in a single song, HVC neurons which project to area RA ($\mathop {\mathrm {\text {HVC}_{\text {RA}}}}\limits$ neurons) fire a precise chain of spikes where each $\mathop {\mathrm {\text {HVC}_{\text {RA}}}}\limits$ neuron fires a burst at a well-defined moment in time^10^. This chain of spikes covers the entire time interval of singing, even during the silent intervals between the different segments (or syllables) of a single song^12^. This chain of spikes is also highly reproducible across singing bouts, with individual spikes displaying sub-millisecond precision^8,10^. For example, the first spike fired in a burst for $\mathop {\mathrm {\text {HVC}_{\text {RA}}}}\limits$ neurons was estimated to have a precision of $0.73\pm 0.3$ ms. At RA, the individual neurons also display highly reproducible spike sequences with sub-millisecond ($0.28 \pm 0.3$ ms) precision^8^. RA serves as one of the final command signals to produce singing through the vocal organ with the hypoglossal nucleus acting as the final relay. The neural architecture at the heart of this reproducible and precise behaviour is a reproducible, precise, and sparse sequence of spikes.

If populations of neurons use precisely timed and reliably emitted spikes to encode stimuli or produce behaviours, how would that impact the ability to decode out these stimuli or behaviours? Indeed, there is substantial computational modelling and analytical results that investigate the accuracy of decoding a stimulus. For example, if the spikes are only emitted as part of a rate code, then prior work shows that we should expect that decoding accuracy only scales with the square-root of the number of spikes fired^13,14^. However, there are also proposed neural circuits that rely on balanced excitatory/inhibitory networks that do not depend on precise and repeatable sequences of spikes yet have decoding errors that decrease linearly with network size^13,15–17^. These networks fire spikes sparsely^15–18^ through a coordinated network structure where a neuron’s time varying inputs are precisely balanced between excitation and inhibition. The precise balance of inputs allows a neuron to compute the decoding error in representing a stimulus, or dynamical system. A spike is only fired when a threshold decision boundary is crossed^15–18^, at which point the error in reproducing a stimulus or dynamical system is reset. These networks are robust to neural heterogeneity^19,20^, neuronal model implementation^17^, and can be learned locally with biologically plausible spike timing-dependent plasticity rules^21–23^. Owing to the error reset mechanism, the spike trains produced have Poisson-like statistics, without explicitly being drawn from a Poisson point process^15–17^.

In this study, we investigate the decoding accuracy of time-series data from precisely timed and regular sequences of spikes. By using synthetically constructed spike-trains and mathematical analysis, we derived sufficient conditions for sequences of spikes to have a qualitative difference over rate codes in the accuracy of decoding time series data. This time series data can represent behaviours, stimuli, or other signals but must be suitably smooth in a mathematical sense. In networks containing perfectly precise and reliable spikes, the root mean squared error (RMSE) of a time-series decoded with a linear decoder decreases linearly with the number of spikes or neurons used in the decoding. This is similar to the aforementioned efficient codes that rely on E/I balance^13,15–17^. We tested these findings in numerical simulations with synthetically generated spike-trains and synthetically generated signals to be decoded. This linear decrease in the RMSE with network size also holds when the spike times are no longer perfectly precise and reliable. However, the spike timing precision and spike emission reliability of a neuron must increase along with the size of a network. The error incurred by approximating the system with spikes can be regarded as the bias in a bias variance decomposition, while the variability in the replay of a signal caused by imprecise or unreliable spikes can be interpreted as the variance. Finally, we discuss how to test the scaling-relationship experimentally in the zebra finch circuit. This circuit exhibits a natural increase in the HVC network size via neurogenesis. We predict that if the decoding accuracy of the stereotypical zebra finch song increases linearly with HVC network size, then so must the precision of HVC spikes.

## Results

Before considering how precise spike times impact the neural code, neural encoding and neural decoding will be defined. As an organism performs a behaviour or perceives a stimulus which is represented with the function of time, *x*(*t*), spikes are emitted as a result of the stimulus or in pursuit of enacting the behaviour. We will assume that the signal *x*(*t*) occupies an interval $t\in [0,T]$ with all spikes generated in the encoding process confined to this interval. As such, the stimulus becomes encoded by some encoding transformation $\varvec{E}$ into a series of spikes which can be represented as a matrix.

1 $$\begin{aligned} \varvec{\tau }= E(x(t)) \end{aligned}$$

Here the vector $\varvec{\tau }$ is an $N\times n_t$ matrix where *N* corresponds to the number of neurons, and $n_t$ is a discretization of time. Thus, we define the matrix $\varvec{\tau }$ as

$$\begin{aligned} \varvec{\tau }_{ij} = {\left\{ \begin{array}{ll}1 &{} \text {if Neuron } \,i \,\text {spiked in } I_j\\ 0 &{} \text {otherwise} \end{array}\right. } \end{aligned}$$

where the $I_j$th time interval corresponds to $I_j = [\frac{jT}{n_t},\frac{(j+1)T}{n_t}]$. We will not explicitly specify how the function *E* generates the encoding matrix $\varvec{\tau }$, but rather consider the conditions on a mapping that allow for accurate decoding. For example, the mapping *E* may generate the same realization of spikes for *x*(*t*) every time yielding reliable and precisely timed spikes. Alternatively, *E* may be stochastic in nature and the spike times may have some noise or “jitter” present, or subsets of the spikes may even fail to be emitted. Thus, the function *E* in (1) defines a basic neural encoding scheme.

Decoding can be thought of as a kind of inverse to Eq. (1). In neural decoding, the goal is to reconstruct *x*(*t*) from $\varvec{\tau }$. In general, inverting Eq. (1) is not possible. However, *x*(*t*) can be approximated with ${\hat{x}}(t)$ and some decoding operator *D*:

2 $$\begin{aligned} {\hat{x}}(t) = D\left( \varvec{\tau }\right) = D(E(x(t)) \end{aligned}$$

One approach to decoding *x*(*t*) is to convolve the spikes with some sort of kernel, *K*(*t*) and then consider the decoder *D* to be a linear transformation, $D = \varvec{\phi }^x$

3 $$\begin{aligned} {\hat{x}}(t)=\sum _{j=1}^N \phi _j^x r_j(t) = \sum _{j=1}^N\sum _{t_{jk}<t}\phi _j^x K(t-t_{jk}) \end{aligned}$$

where $t_{jk}$ is the *j*th spike fired by the *k*th neuron. The Kernel function *K*(*t*) the spikes. Here, we consider the simple exponential filter ($K(t) = \exp (-t/\tau )$ with a filtering time constant of 10 ms. The function $r_j(t)$ is the filtered spike history for neuron *j*. We can interpret $\varvec{r}$ as a temporal basis set. The linear decoder $\varvec{\phi }^x$ transforms the temporal basis set $\varvec{r}(t)$ into the approximation to the decoded signal $({\hat{x}}(t))$.

Typically, the decoder is constructed by using one or a subset of repetitions of the behaviour or stimulus (and spikes) to train the decoder. The trained decoder is later tested with repetitions that were not used to construct the initial decoder.

Neural encoding and decoding raise the question of how accurate can we decode out the function *x*(*t*) with ${\hat{x}}(t)$. The Root-Mean-Squared Error (RMSE) is commonly, although by no means exclusively used as the metric to measure this accuracy^14–17^. For example, if we consider the audio-time series of a zebra finch singing as *x*(*t*), then the RMSE measures the difference between the decoded song (${\hat{x}}(t)$) and the recording with a RMSE of 0 indicating perfect decoding accuracy. The RMSE is defined as:

4 $$\begin{aligned} RMSE = \sqrt{\int _0^T \left( {\hat{x}}(t) - x(t)\right) ^2 \,dt} = \sqrt{MSE} \end{aligned}$$

where MSE denotes the mean-squared-error. With encoding, decoding, and the RMSE of decoding now defined, the question of how accurate can the stimulus *x*(*t*) be reconstructed naturally emerges. More formally, for progressively larger networks ($N\rightarrow \infty$), what is the limiting behaviour of the RMSE?

5 $$\begin{aligned} RMSE = \sqrt{\int _0^T \left( {\hat{x}}(t) - x(t)\right) ^2 \,dt} = O(N^\lambda ), \quad N\rightarrow \infty \end{aligned}$$

Efficient coding networks were proposed in^15–17^ to 1) sparsely fire spikes and 2) accurately and robustly represent signals or internal dynamics all with one-neural architecture. Briefly, these networks use precisely balanced slower connections and faster inhibitory connections which can be learned or pre-configured as a result of an error reducing optimization criterion^15–18,21–23^. These networks are robust to losing neurons, and can accurately produce a desired output. These networks precisely track a desired signal as a decoded representation ${\hat{x}}(t)$. When the error in this representation reaches a critical threshold (or bounding box^18^), a spike is emitted to reset the local error in the representation to 0. The neuron that ultimately fires this spike inhibits the other neurons in the network to maximize the efficiency of the code. Thus, for these networks, the $\lambda$ value in Eq. 5 is -1 and adding more neurons decreases the RMSE linearly with the network size.

As we will demonstrate numerically and analytically, for precisely timed sequences of spikes, such as those fired by $\mathop {\mathrm {\text {HVC}_{\text {RA}}}}\limits$ neurons or pyramidal neurons in RA in the zebra finch, the RMSE also has a $\lambda =-1$ and decreases linearly with the number of neurons:

6 $$\begin{aligned} RMSE = \sqrt{\int _0^T \left( {\hat{x}}(t) - x(t)\right) ^2 \,dt} \propto \frac{1}{N} \quad \text {(Precise spike times and reliable spikes)}. \end{aligned}$$

This is qualitatively different from and in strike contrast to alternate codes. For example, if the timing of spikes is unimportant for the neural code, and only the number of spikes per unit of time (the rate) encodes information, the RMSE decreases with the square root ($\lambda = -1/2$) of the number of neurons^15–17^:

7 $$\begin{aligned} RMSE = \sqrt{\int _0^T \left( {\hat{x}}(t) - x(t)\right) ^2 \,dt} \propto \frac{1}{\sqrt{N}}\quad \text {(Rate Code)}. \end{aligned}$$

### The bias-variance decomposition

To test how the RMSE changes when spikes become imprecisely timed or unreliably emitted, a perturbed decoded approximation ${\tilde{x}}(t)$ is defined as the approximation to *x*(*t*) when the decoder $\varvec{\phi }^x$ is applied to the second set of spikes which suffer from spike failure, where spikes fail to be emitted at their designated time, or spike jitter, where the spike time of a spike is randomly perturbed. Then the MSE can be decomposed as

8 $$\begin{aligned} MSE= & {} \int _0^T \left( {\tilde{x}}(t) - x(t)\right) ^2\,dt = \Vert {\tilde{x}} - x \Vert ^2 \nonumber \\= & {} \Vert {\tilde{x}} -{\hat{x}} + {\hat{x}} - x \Vert ^2 \nonumber \\\le & {} (\Vert {\tilde{x}} - {\hat{x}}\Vert + \Vert {\hat{x}} -x\Vert )^2 = \Vert {\hat{x}} -x\Vert ^2+ \Vert {\tilde{x}} - {\hat{x}}\Vert ^2 + 2 \Vert {\hat{x}} -x\Vert \Vert {\tilde{x}} - {\hat{x}}\Vert \end{aligned}$$

9 $$\begin{aligned}= & {} Bias^2 + Variance + 2 Bias \cdot Std \end{aligned}$$

where the square of the RMSE is the mean squared error (MSE) and $\Vert \Vert$ is the standard $L_2$ function norm defined over [0, *T*]. The first term in Eq. (8) can be interpreted as the bias in the bias-variance trade-off, while the scond term is the variance (square of the standard deviation). The first term quantifies how well we can approximate *x*(*t*) with a spike train, while the second term is the error incurred in assuming that the spike train is perfectly repeatable.

### Precise and reliable spike times lead to a linear decrease in the root mean squared error (RMSE) with increasing network sizes

**Figure 1:**
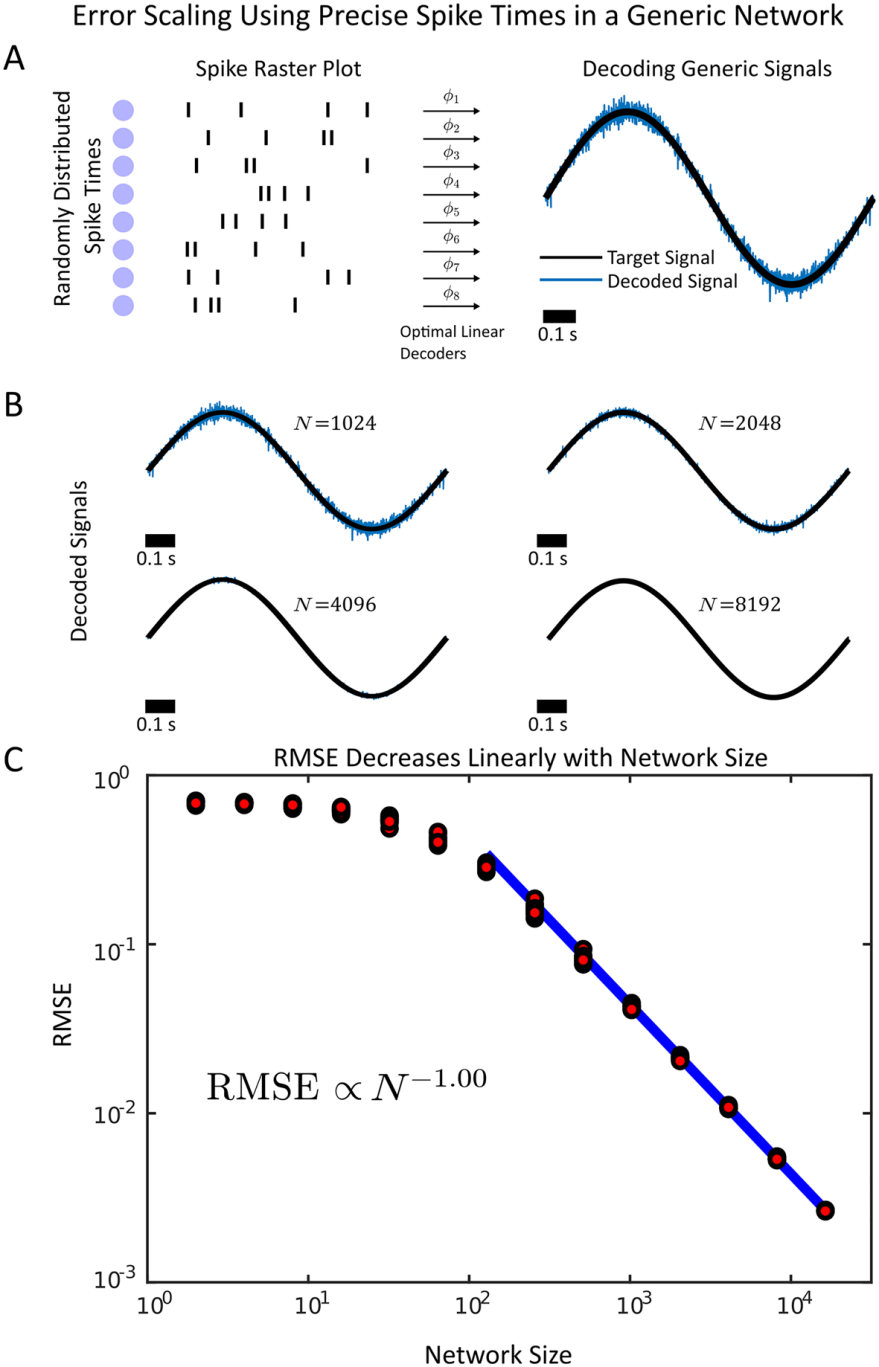
(**A**) (Left) A diagram of a randomly generated spike sequence used to decode a time series with optimal linear decoders. A decoded signal (blue) along with the target supervisor (black) is shown in the right. The signal is decoded using $N=1024$ neurons. (**B**) Decoding the time series with larger networks. The spikes generated are drawn with the same statistics as in (A) and filtered identically. The signal is decoded for progressively larger networks ($N=2^{10}$ to $N=2^{14}$). (**C**) The loglog plot of the RMSE vs network size. The RMSE decreases linearly with the network size. The linear fit was performed to the mean of the 10 realizations from $N=2^{10}$ to $N=2^{14}$. The largest five.

To investigate how spike timing precision and spike emission reliability impact decoding accuracy, we constructed synthetic spike trains where each neuron fires a variable number of spikes. These spikes were generated randomly from a Poisson distribution, with the same realization of spikes drawn on each trial. Further, the spikes are reliable and precisely timed from trial to trial as they are drawn from the same realization, however they can be easily and independently perturbed in subsequent trials (Fig. 1A-B). We constructed optimal linear decoders that could decode out a target signal, in this case, a simple sinusoidal oscillator with a period of 1 Hz (Fig. 1A). We found that the RMSE decreased linearly (RMSE $\propto N^{-1.00}$) with the network size for sufficiently large networks. Thus, if spike times are perfectly precise, they can be used to decode out a continuous signal with $N^{-1}$ RMSE scaling. The spikes do not need to explicitly encode features of the signal, but merely tile time with sufficiently large numbers. This is an identical RMSE scaling to efficient codes^15–17^ and superior to the error scaling achieved by using firing rates (RMSE $\propto N^{-1/2}$).

Next, we investigated for what general conditions this result would hold (Supplementary Material S1 and Fig. S1). We found that the linear decrease in the RMSE with network size is a general, mathematical result that occurs so long as the time series that the spikes must approximate is sufficiently smooth (differentiable). This criterion can be relaxed to the function being Lipschitz continuous (Supplementary Fig. S1). The spikes need not be ordered into a chain or generated randomly as above but must be precise from trial-to-trial and sufficiently dense in time (Supplementary Material S1). Under these conditions, the efficient coding error scaling $RMSE \propto N^{-1}$ is likely to be achieved for sufficiently large network sizes. Further, the spikes can overlap and interfere with each other extensively (Supplementary Fig. S2) and need not be sparsely distributed or orthogonal. As long as the spikes are precisely timed, the RMSE will still decrease linearly with the network size (Supplementary Fig. S2) under the smoothness conditions considered in the Supplementary Material. Alternatively stated, in the case of perfectly timed and perfectly reliable spikes and a suitably smooth supervisor, the bias term in Eq. (8) decreases linearly with the network size, while the variance is 0 as the spikes contain no degree of imprecision or unreliability.

### The precision of spike times and reliability of spikes must increase with the network size to preserve RMSE $\propto N^{-1}$ scaling

While spike times in the zebra finch $\mathop {\mathrm {\text {HVC}_{\text {RA}}}}\limits$ neurons and RA neurons are precise, they are not perfectly precise. From trial-to-trial, the spike times often display sub-millisecond levels of jitter. This is true for neurons in both the HVC and RA^8,10^ where the jitter is *O*(0.1) ms (depending on the neuronal subtype). Further, neurons can also fail in firing spikes owing to unreliable synapses^24^. Thus, we considered just how precise and reliable spikes should be when increasing the network size to maintain a linear decrease in the RMSE.

**Figure 2:**
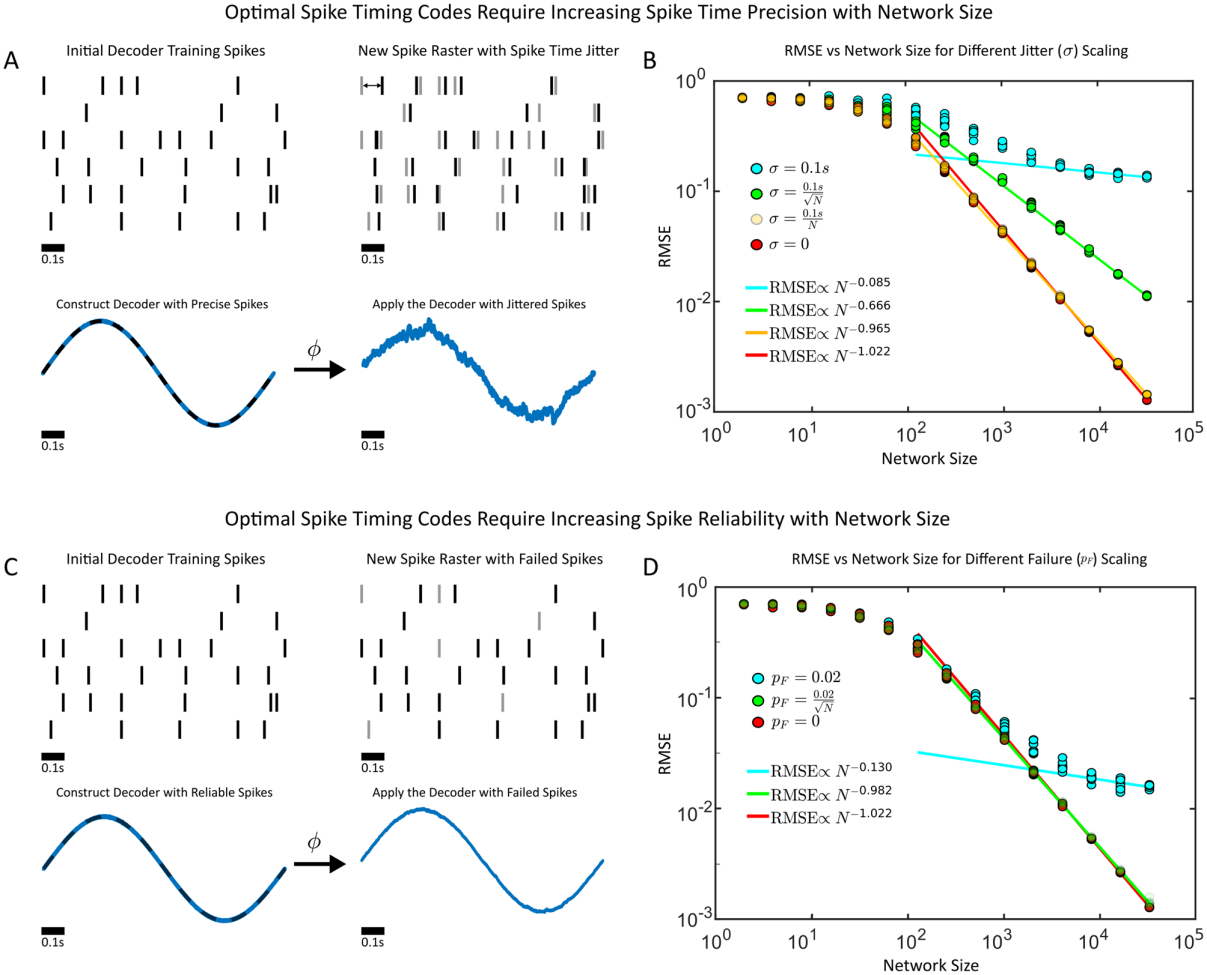
(**A**) Synthetic spike trains are generated and used to train an optimal decoder on a simple sinusoidal oscillator with a period of 1 second. The spike trains are then jittered and the decoder is reapplied, with the resulting root mean squared error (RMSE) measured. (Top) A cartoon of the spike generation protocol: the spikes are first generated to train a decoder. A new spike raster is then generated by jittering the spikes with the decoder reapplied. A sample curve for $N=2^{14}$ shown where the spikes are jittered randomly with mean 0 and standard deviation $\sigma =0.1$s. The supervisor is overlayed (black dashed line) during training. (**B**) The RMSE as a function of the network sized (*N*) for differing jitter amounts $\sigma$. The jitter is either fixed at 0.1 seconds (blue), decreases like the square-root of the network size (green), linearly with the network size (orange) or remains fixed with network size. (**C**) Synthetic spike trains are generated and decoders are trained as in (A), only now a random subset of spikes fail with $p_F$ denoting the spike failure probability. A $p_F=1$ implies that all spikes fail, while a $p_F =0$ implies that the spikes are perfectly reliable. (Top) A cartoon of the spike generation protocol: the spikes are first generated to train a decoder. A new spike raster is then generated by dropping spikes randomly with probability $p_F$ with the decoder reapplied after. A sample curve for $N=2^{14}$ shown where the spikes are dropped randomly with $p_F = 0.02$. The supervisor is overlayed (black-dashed) line during training. (**D**) The RMSE as a function of the network sized (*N*) for differing spike failure amounts $p_F$.

First, we considered spike-jitter from trial-to-trial by constructing a decoder with initially precise spikes. Then, we perturbed the spike times randomly and reapplied the decoder on subsequent trials, and measured the RMSE (Fig. 2A). We found that if the standard deviation of the jitter ($\sigma$) was fixed, the $N^{-1}$ error scaling in the RMSE was no longer present (RMSE $\propto N^{-0.085}$). This was numerically confirmed for trained optimal linear decoders (Fig. 2). The sub-linear decrease in the RMSE was also confirmed for other fixed values of the standard deviation of the spike jitter (Supplementary Fig. S3).

If a fixed amount of timing imprecision would destroy the linear decrease in the RMSE, could populations of neurons increase their spike timing precision with network size to restore it? To investigate this hypothesis, we considered the possibility that spikes might become more precisely timed with increasing network sizes by having the jitter decrease with larger networks (either $\sigma \propto \sqrt{N}^{-1}$ or $\sigma \propto N^{-1}$, Fig. 2B). We found that if the spike times became more precise linearly ($\sigma \propto N^{-1}$) with the network size, this was sufficient to restore the linear decrease in the RMSE with network size (RMSE $\propto N^{-0.965}$).

Next, we considered the case where spikes might abruptly fail (Fig. 2C,D). We again found that if the probability of spike failure ($p_F$) was fixed and independent of the network size, the linear decrease in the RMSE with increasing network size would no longer be present (RMSE $\propto N^{-0.130}$). However, once again, if the spikes are less likely to fail with larger networks ($p_F \propto \sqrt{N}^{-1}$), the linear decrease in the RMSE would be restored (RMSE $\propto N^{-1.022}$). These results were qualitatively and quantitatively similar to the case where spikes were randomly emitted (rather than randomly failed, Supplementary Fig. S4). Thus, spike timing imprecision or spike-failure are themselves not sufficient to eliminate the linear decrease in the RMSE, but the spikes can become more reliable and more precisely timed with progressively larger network sizes to maintain RMSE $\propto N^{-1}$ scaling. The probability of a spike failing can decrease with the square-root of the network size while the standard deviation of spike jitter should decrease linearly with the network size as a sufficient condition to preserve a linear decrease in RMSE scaling.

The impacts of spike timing imprecision, and spike emission reliability were also explored analytically, albeit with simplifying assumptions for tractability (Supplementary Material S2). Indeed, in the bias-variance decomposition, sufficient (but not necessary) conditions were determined to restrict the variance to scale like $N^{-2}$, which leads to the RMSE scaling like $\propto N^{-1}$. First, we considered the case of a simple neuron-specific jitter to a filtered, but differentiable, spike train. This would be the case for example if the spikes were filtered with Gaussian filters where every spike for a single neuron is jittered by the same amount, which differs from the jitter applied to the other neurons’ spikes. If the sum of the absolute decoders, given by $\sum _{j=1}^N |\phi _j^{x(t)}|$, is bounded for $N\rightarrow \infty$, then $\sigma \propto N^{-1}$ is a sufficient condition for RMSE$\propto N^{-1}$ as $N\rightarrow \infty$. For the case of failing spikes, we considered evenly distributed spikes with box filters. In this case, each spike does not have any redundancy in the form of overlapping spikes from other neurons to aid in the coding of a signal, as all the spike trains are orthogonal. In this scenario, it is sufficient for the spike failure rate to scale with $p_F \propto N^{-2}$, which differs radically from the numerical results of $p_F \propto \sqrt{N}^{-1}$ to maintain RMSE$\propto N^{-1}$. This discrepancy where the numerical simulations result in looser restrictions on the probability of spike failure may be due to the simplifying assumption of non-redundant spiking in the analytical derivation for tractability. To investigate this more thoroughly, the simulations in Fig. 2 were conducted with larger initial $p_F$ ($p_F = 0.05$ and $p_F = 0.5$ Supplementary Fig. S3). In the latter case, we did find that RMSE $\propto -0.703$ when the $p_F \propto \frac{0.5}{\sqrt{N}}$. This may imply that the decrease in RMSE with $p_F$ is dependent on the initial $p_F$ with larger initial failure probabilities overwhelming any redundancy that additional spikes provides as the network becomes larger, or alternatively, the sub-linear decrease in RMSE with network size for larger $p_F$ may be a numerical transient.

**Figure 3:**
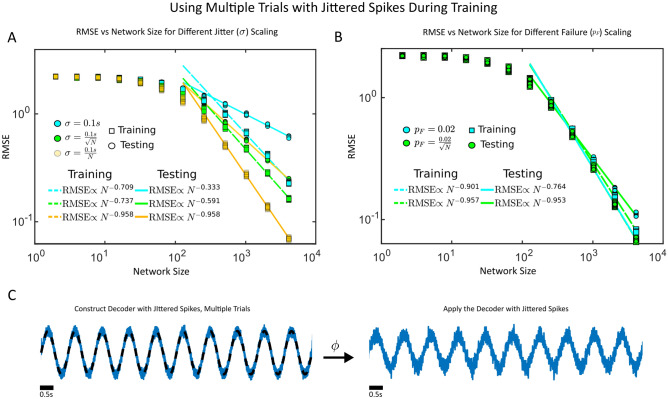
(**A**) The impact of multiple trials on RMSE scaling. Randomly generated spike sequences with imprecise and reliable spike times are used to linearly decode a sinusoidal oscillator with a period of 1 second. The decoder is trained with 10 repetitions of a spike train with jittered spikes and tested with another 10 repetitions to measure the RMSE. A total of 10 realizations of this procedure are used for each *N*. The spike jitter amount decreases linearly with *N* (yellow), with the square-root of *N* (green), or stays constant (teal). (**B**) Randomly generated spike sequences with precise spike times but unreliable spikes are used to linearly decode a generic a sinusoidal oscillator with a period of 1 second. The decoder is trained with 10 repetitions of a spike train with failed spikes and tested with another 10 repetitions to measure the RMSE. A total of 10 realizations of this procedure are used for each *N*. (**C**) The target time series (black dashed lines) and the decoded estimate (blue). The decoder is reapplied in the left on a test trial of jittered spikes. The decoded signal is trained and tested in (**C**) with $N=2^{12}$ neurons and a jitter of $\sigma = 0.1$ s. Note that for (**A**) and (**B**), the maximum network size considered was $N=2^{12}$.

In Figs. 1 and 2, the decoders were trained with a single repetition of the supervisor and spike train. We tested the hypothesis that more repetitions would improve RMSE scaling with *N* by training a network with 10 trials of imprecise (Fig. 3A,C) or unreliable spikes (Fig. 3B). The linear decrease in RMSE with increasing network size was only maintained under identical scaling conditions as in Fig. 2 for the spike jitter (RMSE $\propto N^{-0.958}$) where the standard deviation decreased linearly with network size, and spike failure (RMSE $\propto N^{-0.953}$) where the probability of spike failure decreased with the square root of the network size. However, there was a notable improvement in RMSE scaling when using multiple trials when the probability of spike failure was constant ($p_F = 0.02$). When multiple trials were used, RMSE $\propto N^{-0.764}$ while a single training trial lead to RMSE $\propto N^{-0.243}$. It is possible that with more training trials, the exponent would shift further closer to $-1$. Thus, to preserve the linear decrease in RMSE with network size, even when multiple trials are used to train a decoder, the spikes should still become more reliably emitted and more precisely timed with network size to maintain a linear decrease in the RMSE with network size.

### The impacts of supervisor complexity and smoothness on linear RMSE scaling

**Figure 4:**
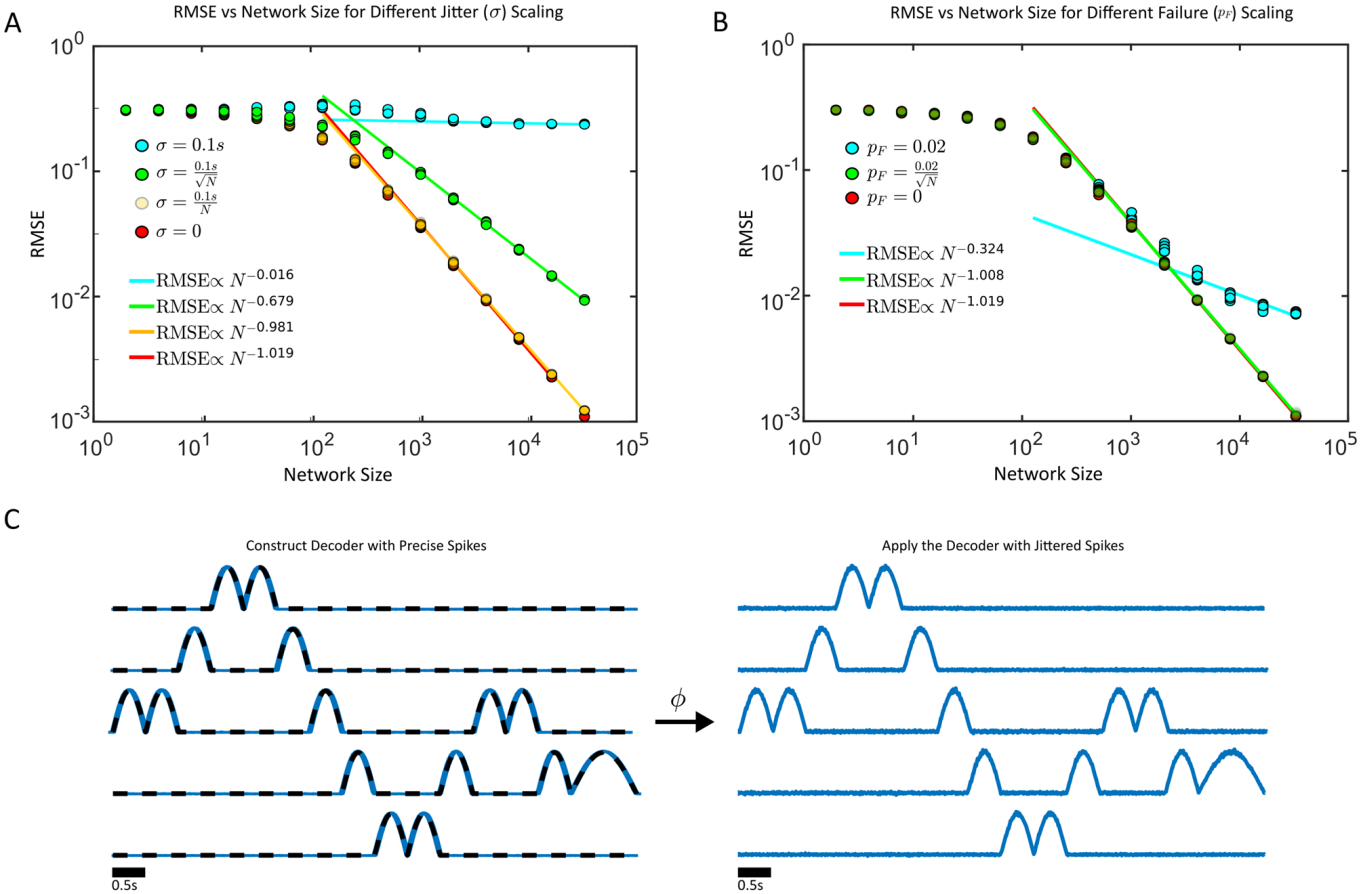
(**A**) Imprecise but reliable spikes are used to decode a 5-dimensional sequence of pulses that replicate the song “Ode to Joy”. The root mean squared error is plotted versus the network size. The spike jitter amount decreases linearly with *N* (yellow), with the square-root of *N* (green), or stays constant at $\sigma = 0.1$ s (teal), or stays constant at 0 (red). (**B**) Precisely timed but unreliable spikes with a probability of spike failure $p_F$ are used to decode Ode to Joy. The spike failure probability either decreases with the square root of the network size (green), or is fixed at $p_F = 0.02$ (teal), or is fixed to 0 (red). (**C**) The decoded Ode to Joy signal at $N=2^{15}$ with $\sigma \propto \sqrt{N}^{-1}$ (right). The jitterless signal (blue) and the supervisor (black) are shown in the left. The pulse width denotes either a quarter note or a half note, while the pulse dimension denotes the specific note.

The decoded signals thus far were simple smooth functions (e.g., sinusoidal inputs). We sought to determine how increasing the potential complexity of a supervisor would impact the scaling of the RMSE. First, we considered a multi-dimensional supervisor consisting of a sequence of pulses of variable duration (Fig. 4). The pulses represent different notes for the song “Ode to Joy” (see^14^ for further details). We found that precisely timed and reliably emitted spikes still lead to a linear decrease in the RMSE with network size (RMSE $\propto N^{-1.019}$, Fig. 4A). However, if the spikes are imprecise, the standard deviation of the spike jitter must decrease linearly with the network size to preserve the linear error scaling of the RMSE (RMSE $\propto N^{-0.981}$). If the spikes are unreliable, the probability of spike failure must also decrease with the square root of the network size to preserve the linear error scaling of the RMSE (RMSE $\propto N^{-1.008}$). Collectively, these results imply that the linear decrease of the RMSE with network size is robust to different supervisors as the RMSE scaling was identical to the results in Figs. 1, 2 and 3.

**Figure 5:**
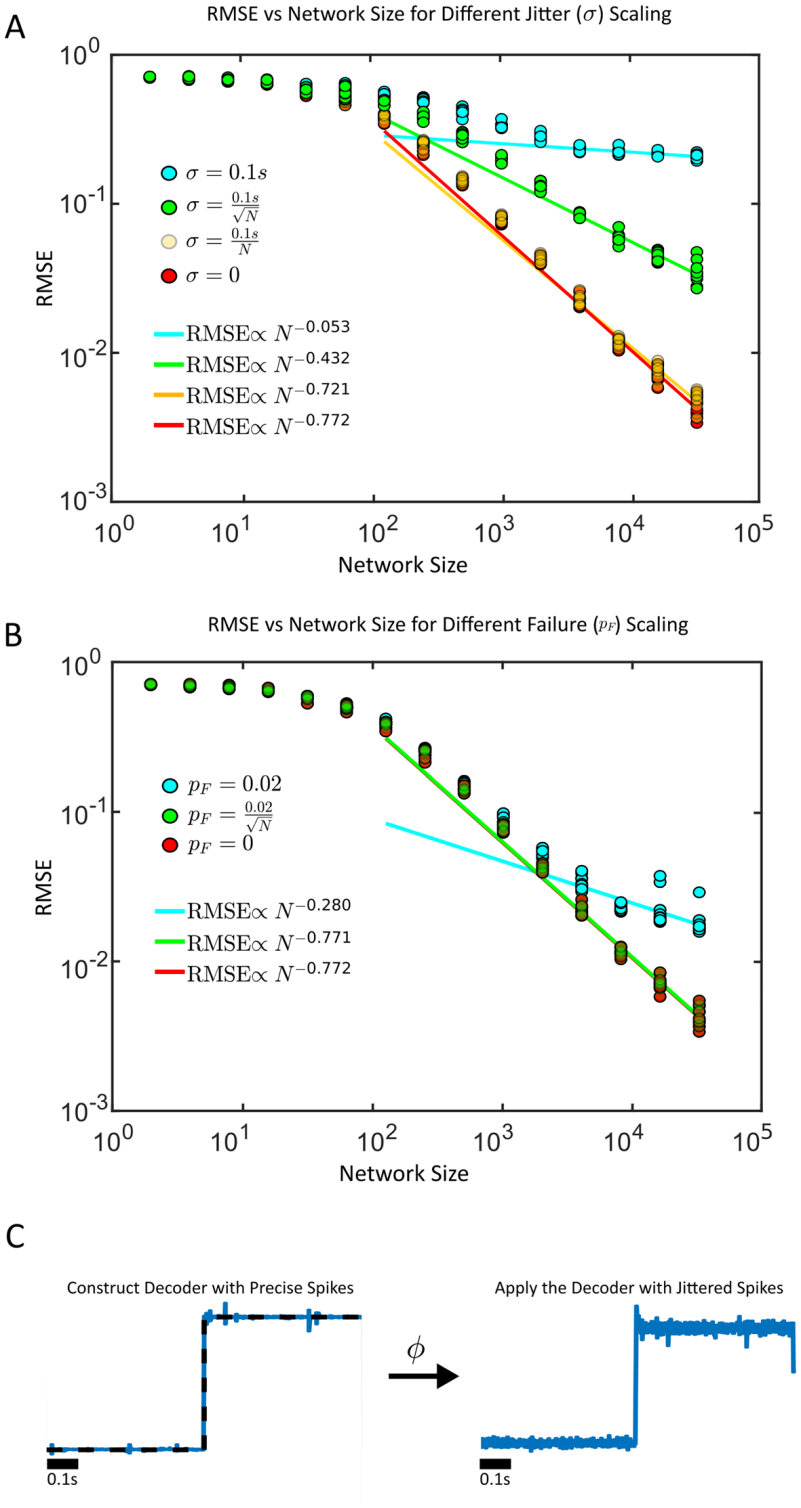
(**A**) Imprecise but reliable spikes are used to decode a nonsmooth function $f(t) = \text {sign}(t-0.5)$. The root mean squared error is plotted versus the network size. The spike jitter amount decreases linearly with *N* (yellow), with the square-root of *N* (green), or stays constant at $\sigma = 0.1$ s (teal), or stays constant at $\sigma = 0$ (red). (**B**) Precisely timed but unreliable spikes with a probability of spike failure $p_F$ are used to decode the sign function. The spike failure probability either decreases with the square root of the network size (green), or is fixed at $p_F = 0.02$ (teal), or is fixed to $p_F=0$ (red). (**C**) The decoded sign function signal at $N=2^{15}$ with $\sigma \propto \sqrt{N}^{-1}$ (right). The jitterless signal (blue) and the supervisor (black) are shown in the left.

Next, we considered if the continuity of the decoded signal was critical for the linear decrease in RMSE with larger network sizes (Fig. 5). To that end, we utilized a simple but non-smooth signal (the sign function) that exhibits a discontinuity (Fig. 5A–C). Surprisingly, even when the spikes are both reliable and precise, the RMSE did not decrease linearly with the network size, but at a slower rate (RMSE $\propto N^{-0.772}$, Fig. 5A–B). This signal is also more poorly decoded when the spikes suffer from spike jitter or failure, however the slower RMSE scaling is restored when the standard deviation of the jitter decreases linearly with the network size (RMSE $\propto N^{-0.721}$, or when the spike failure rate decreases with the square root of the network size (RMSE $\propto N^{-0.771}$). The source of the discontinuity is where the magnitude of the error is the largest (Supplementary Fig. 5). We note that the point of discontinuity also explicitly breaks the smoothness conditions (and more generally the Lipschitz condition) required for RMSE $\propto N^{-1}$ scaling in the derivation (Supplementary Material S1). Further, we show that approximating a nonsmooth step function will change the bias scaling in the bias-variance decomposition to bias $\propto N^{-1/2}$ (Supplementary Material S2). This was derived analytically under simplifying assumptions about the nature of the spike train (evenly distributed single spikes) and filtering (box-filtering) which differs from the more realistic numerical examples considered. Nevertheless, the smoothness of the supervisor impacts the RMSE scaling via the bias term in the bias-variance decomposition.

### Testing the RMSE versus network size in the zebra finch

**Figure 6:**
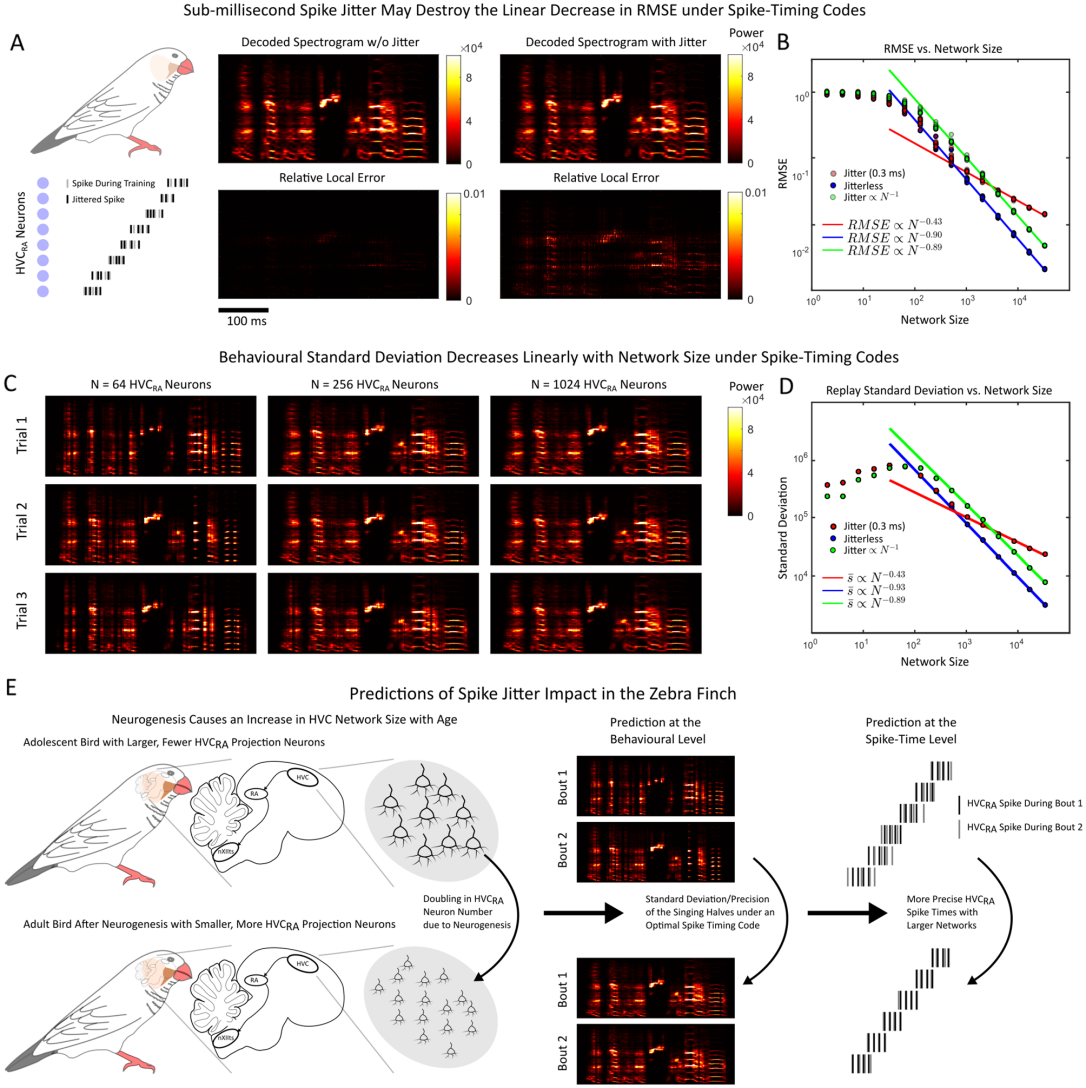
(**A**) Synthetic $\mathop {\mathrm {\text {HVC}_{\text {RA}}}}\limits$ synfire chain spike sequences are created and used to train a decoder to decode out a sample spectrogram. These spikes are generated as bursts of 4 spikes per cell. The time the initial spike in a burst is randomly sampled from a uniform distribution on [0, 0.88]s. These spikes are then jittered with varying amounts of jitter with the decoder reapplied and the RMSE measured. (**B**) Root Mean Squared Error (RMSE) between the song spectrogram and the decoded output as a function of the network size (*N*) with varying amounts of jitter. Sub-millisecond jitter can destroy the $\approx N^{-1}$ scaling in the RMSE. The standard deviation of the jitter was taken to be $\sigma = 0$ (blue), $\sigma = 0.3$ ms (red), and $\sigma = \frac{1s}{N}$ (green). (**C**) Sample decoded spectrograms for different values of the network size for the fixed jitter $\sigma = 0.3$ ms case. (**D**) The spectrogram variance ${\bar{s}}^2$ (see Materials and Methods) displays identical scaling relationships as the RMSE. The standard deviation of the jitter was taken to be $\sigma = 0$ (blue), $\sigma = 0.3$ ms (red), and $\sigma = \frac{1s}{N}$ (green). (**E**) The prediction of linear RMSE scaling in the zebra finch circuit. An increase in network size (in this case a doubling) due to neurogenesis leads to a proportional increase in behavioural stereotypy/precision (or decrease in the behavioural standard deviation), and an increase in the precision of spike times in $\mathop {\mathrm {\text {HVC}_{\text {RA}}}}\limits$ neurons.

We investigated what the impact of precise spike times would have on the RMSE of a decoded behaviour: the song produced by the zebra finch. We first constructed a simplified model of the zebra finch circuit with synthetically constructed spike trains that mimic the statistics of $\mathop {\mathrm {\text {HVC}_{\text {RA}}}}\limits$ neurons (Fig. 6A). In particular, each $\mathop {\mathrm {\text {HVC}_{\text {RA}}}}\limits$ neuron fires a single burst of spikes, and the bursts are sampled from a uniform distribution over the entire duration of the song. A decoder ($\varvec{\phi }^x$) is then constructed using a zebra finch song recording (see Materials and Methods,^14^, Fig. 6B). We considered three scenarios: a fixed jitter at $\sigma =0.3$ ms a jitterless network with $\sigma =0$ ms, and a network for which the jitter would decrease with N with $\sigma = \frac{1 s}{N}$. Note that we took a larger jitter value for small *N* so that the overall jitter in the latter case would be comparable to 0.3 ms for large *N*. We found that larger networks (increasing *N*) of $\mathop {\mathrm {\text {HVC}_{\text {RA}}}}\limits$ neurons resulted in more accurate decoding (Fig. 6B). For sufficiently large networks, the RMSE decreased approximately linearly when the jitter was either not present (RMSE $\propto N^{-0.9}$), or decreased linearly with the network size (RMSE $\propto N^{-0.89}$). We remark that the RMSE scaling exponent is slightly larger than -1, which may be due to discontinuities or rapid signal changes in the spectrogram (Supplementary Material S2). If the jitter was fixed at $\sigma = 0.3$ ms, the RMSE decreased sublinearly with network size (RMSE $\propto N^{-0.43}$).

While the RMSE is important for computational studies, it is difficult for an experimental neuroscientist to actually utilize and measure for a simple reason: they do not have access to the signal a circuit intends to represent, only the output of the circuit itself. Indeed, the RMSE represents the accuracy of a behaviour once that behaviour is known. Thus, we tested whether the precision of a behaviour also increased with network size by measuring the standard deviation of the decoded spectrograms (Fig. 6C–D). We found that the behavioural variability (see Materials and Methods for definition) of the spectrograms displayed similar scaling relationships (slopes of -0.43, -0.93, and -0.89) as the RMSE (slopes of -0.43, -0.9, and -0.89) for all conditions ($\sigma = 0.3$ ms, $\sigma = 0$ ms, $\sigma \propto N^{-1}$). This result allows an experimentalist to record a small number of repetitions (10 in this case) of animal singing, and use the stereotypy of a song as a proxy for the RMSE in measuring these scaling relationships.

## Discussion

Precisely timed spikes have been hypothesized to somehow encode information^25–34^. Here, we explored the hypothesis that spike timing precision and spike emission reliability are specifically used to improve the accuracy of decoding a time series, as measured by the root mean squared error (RMSE). The RMSE in decoding any behaviour or sensory stimulus decreases linearly with the network size when using an optimal linear decoder with precisely timed and reliable spikes. This is similar to efficient coding circuits^15–18,21–23^, or the transition between rate and timing codes considered by others^34^. We found that this linear decrease in RMSE was robust to different supervisors, and different distributions of spikes, so long as the spikes were precise and reliable, and sufficiently smooth. If the standard deviation in the spike times remained fixed with network size, the linear decrease in the RMSE with network size would be destroyed. If, however, spike emission reliability and spike timing precision increased with the network size, the linear decrease in RMSE with network size would be retained. The standard deviation of the spike jitter had to decrease linearly with network size while the probability of spike failure had to decrease with the square-root of the network size for a linear decrease in the RMSE with network size to be maintained.

The sub-linear increase in the RMSE with network size can in many of the cases considered here, be attributed to the variance in the bias-variance decomposition of the RMSE. In both analytical results, and numerical simulations, we found that for smooth supervisors, the bias term in the RMSE would decrease linearly with the network size, while the variance would decrease sub-linearly unless the variance could be controlled to also decrease with square of the network size by firing spikes more reliably and more precisely. We remark that as the bias and the variance can scale at different rates, accurate empirical estimates of the asymptotic scaling relationship in the RMSE may require larger network sizes than those considered here.

The numerical and analytical exploration of the coding accuracy of a stimulus considered here can inform reservoir computing approaches with spikes^14,35–37^, where the learning is primarily in the readout weights, and more generally, neuromorphic computing, where spiking neurons are used in hardware implementations of neural circuits^3,4^. Our results show that there are diminishing returns in adding either more spikes or more neurons to improve decoding, unless the spike timing reliability and spike failure rates can be suitably controlled with larger network sizes. These results show that hardware resources should be dedicated to improving the precision of spikes or their reliability, instead of increasing the network size for networks that are already large. This is in line with recent results that examine network size versus error rates in non-spiking networks^39^.

The zebra finch is well positioned to test hypotheses about how the precision in reproducing behaviours is determined by the number of neurons controlling said behaviour. First, the circuit controls a stereotyped behaviour that is readily elicited and easily recorded with a microphone in head-fixed animals. Second, new $\mathop {\mathrm {\text {HVC}_{\text {RA}}}}\limits$ neurons naturally form through the process of neurogenesis^40–45^ in HVC, and roughly double in number from an average of around 40, 000 $\mathop {\mathrm {\text {HVC}_{\text {RA}}}}\limits$ neurons in the first year of life, to around 80, 000 $\mathop {\mathrm {\text {HVC}_{\text {RA}}}}\limits$ neurons by year 11 (see Fig. 2 in^40^, Fig. 6). Our work predicts that singing bouts will become more regular or equivalently, decrease in their variance linearly with the size of the network if the spikes also increase in precision and reliability. The precise trend comparing the behavioural precision with the network size of $\mathop {\mathrm {\text {HVC}_{\text {RA}}}}\limits$ neurons has not been currently ascertained. However, there are a pair of studies that separately imply that the total number of $\mathop {\mathrm {\text {HVC}_{\text {RA}}}}\limits$ neurons increases with age^40^ and that the singing precision also increases with age^44^. However, the authors in^44^ postulated that the increase in behavioural precision was inversely related to the rate of neurogenesis, rather than the overall number of $\mathop {\mathrm {\text {HVC}_{\text {RA}}}}\limits$ neurons.

As an alternative to naturally allowing the $\mathop {\mathrm {\text {HVC}_{\text {RA}}}}\limits$ neurons to increase in number with neurogenesis, one can selectively inhibit $\mathop {\mathrm {\text {HVC}_{\text {RA}}}}\limits$ neurons with optogenetics or ablate a fixed proportion of the HVC nucleus entirely^11^. This would deactivate a random subset of $\mathop {\mathrm {\text {HVC}_{\text {RA}}}}\limits$ neurons. After the animal is given a suitable amount of time to recover and relearn from the deactivation of said neurons, the resulting circuit should produce the identical song, but with fewer $\mathop {\mathrm {\text {HVC}_{\text {RA}}}}\limits$ neurons. This recovery period should also be smaller than the (very slow) timescale of $\mathop {\mathrm {\text {HVC}_{\text {RA}}}}\limits$ neurogenesis. Here, we would expect the behavioural precision to decrease linearly with the proportion of neurons deactivated. We note that selective perturbations in efficient codes were also studied in^18^ (see Figs. 4, 5, 6), and the impacts on the bounding box of an efficient code. We also note that it is possible that a trained decoder would be able to predict the resulting changes to the song after perturbation, or potentially predict the natural fluctuations of the song with a sufficiently large training sample.

Further, we remark that precise and reliably emitted spikes are sufficient to generate a linear decrease of the RMSE with network size, however, this is not the only possibility. In fact, linear error scaling was previously predicted under efficient spike time coding schemes^15–23^. Here, neurons explicitly code the error in the representation of a stimulus, behaviour, or internal dynamic state with their voltages. When the error reaches a critical threshold, a neuron fires a spike to explicitly reset the error to 0. The end result is a network that also produces a linear scaling of the RMSE with network size. However, owing to the explicit error correction mechanism in these circuits, the spike times for individual neurons are not precisely timed across multiple trials, but are precisely timed to reset the error in a representation^15–17,21^. Here, we suggest an alternate mechanism for $N^{-1}$ error scaling in a circuit: stabilize the spike times and spike emission reliability for larger networks.

This theoretical work demonstrates the sufficient conditions for a neural circuit to qualitatively improve the decoding of a stimulus or behaviour with precisely timed and reliably emitted spikes. Under sufficiently precise and reliable spiking, a network can double its size to double the accuracy of encoding/decoding a stimulus. However, said network must also double the precision of all spikes in the network, and increase the reliability with which spikes are emitted, but by less than double the network size.

## Materials and methods

### Generating synthetic spike trains

Each network considered here constitutes *N* synthetically generated spike trains, with each spike train representing a single neuron. Thus, the synthetic spike times, $t_{ji}$ which corresponds to the *i*th spike fired by the *j*th neuron that could have their spike timing precision and spike emission reliability controlled. These spikes were then filtered with a synaptic filter:

10 $$\begin{aligned} r_j(t) = \sum _{t_{ji}<t} K(t-t_{ji}). \end{aligned}$$

The filtering or Kernel function, *K*(*t*) was taken to be a single-exponential filter for all numerical simulations:

11 $$\begin{aligned} K(t) = \exp \left( -\frac{t}{\tau _s}\right) \end{aligned}$$

where $\tau _s = 10$ ms was used as the synaptic filter, approximating the time constant of AMPA synapses. The generation of spike times $t_{ji}$ is described in greater detail below.

#### $\mathop {\mathrm {\text {HVC}_{\text {RA}}}}\limits$ spike train

Each $\mathop {\mathrm {\text {HVC}_{\text {RA}}}}\limits$ neuron consisted of a synthetically generated spike train consisting of 4 spikes in an isolated burst. The spikes were separated by 3 ms inter-spike-intervals, with the initial spike time drawn from a uniform distribution on the interval [0, *T*], where $T=0.88$ seconds is the duration of the song-recording. To vary the network size *N*, the networks were successively doubled in size from $N=2^1$ to $N= 2^{15} = 32768$. The networks in Fig. 6 were simulated 10 times for a fixed *N*, with different seeds in each network, thereby generating different starting times for each $\mathop {\mathrm {\text {HVC}_{\text {RA}}}}\limits$ burst.

To jitter the spikes, each spike time $t_{ji}$ was randomly perturbed by $\epsilon _{ji}$, where $\epsilon _{ji}$ was a normally distributed random variable with mean 0, and standard deviation, $\sigma$. The values of $\sigma$ vary within figures with perfectly precise spikes ($\sigma = 0$ milliseconds), spikes with sub-millisecond precision ($\sigma = 0.3$ milliseconds, Fig. 6), and spikes that become increasingly precise with larger network sizes ($\sigma \propto \frac{1}{N}$).

#### Poisson generated spike trains

The randomly generated spikes in Figs. 1, 2, 3, 4 and 5 were generated from a Poisson process with firing rate $\nu = 2$ Hz, for successively larger networks which were doubled in size from $N=2^1$ to $N=2^{14}$. As in the songbird example, the spikes were perturbed in time with a normally distributed random variable with mean 0 and standard deviation $\sigma$. To implement spike-failure, decoders were first constructed with all spikes generated. These decoders remained fixed, even after the spike times or spike reliability is altered. Then, the fixed decoders were applied to the same spike trains but with each spike having a probability of $p_F$ of failure. To implement spike interference, spikes were added randomly to neurons in time with probability $p_I$. The total number of spikes added was $p_I n_{spikes}$ where $n_{spikes}$ was the number of spikes generated in the nominal, reliable spiking case. The added spikes were randomly distributed from a uniform distribution in time, and across neurons.

### Constructing linear decoders

Linear decoders were constructed by first generating filtered spike-trains that contained no jitter or failure. Then the solution to the optimal linear decoder for a given signal *x*(*t*) is:

12 $$\begin{aligned} \varvec{\phi }^x = \left( \int _0^T \varvec{r}(t)\varvec{r}(t)^T\,dt \right) ^{-1} \int _0^T \varvec{r}(t) x(t)\,dt \end{aligned}$$

The decoder can then be applied to new spike trains to decode ${\hat{x}}(t)$ when these new spikes display either imprecision in their spike times or the failure of spike emission relative to the initial spike train used to construct $\varvec{\phi }^x$. For the simple sinusoidal example, $x(t) = \sin (2\pi t)$, with the approximation ${\hat{x}}(t)$ given by:

13 $$\begin{aligned} {\hat{x}}(t) = \sum _{j=1}^N \phi _j r_j(t), \end{aligned}$$

while for the spectrogram, each frequency component of the song has its own decoder:

14 $$\begin{aligned} \varvec{\phi }^{p(f)} = \left( \int _0^T \varvec{r}(t)\varvec{r}(t)^T\,dt \right) ^{-1} \int _0^T \varvec{r}(t) p(f,t)\,dt \end{aligned}$$

The term *p*(*f*, *t*) is the power of the frequency component *f* at time *t* in the spectrogram, as defined in^14^, while the decoder component $\phi ^{p(f)}$ is the optimal decoder for the power frequency component *p*(*f*, *t*), yielding the approximation:

$$\begin{aligned} {\hat{p}}(f,t) = \sum _{j=1}^N \phi _j^{p(f)}r_j(t) \end{aligned}$$

The frequency range considered varies from a low of $f = 172.27 Hz$ to a high of $f = 10$ kHz, discretized with 229 evenly distributed points, thereby making $\phi ^{p(f)}$ a $N\times 229$ dimensional decoder.

### Measuring the root mean-squared error

The RMSE for the decoded oscillators in Figs. 2F–H and 3 is given by:

15 $$\begin{aligned} RMSE = \sqrt{\int _0^1 \left( {\hat{x}}(t) -x(t)\right) ^2 \,dt} \end{aligned}$$

where $x(t) = \sin (2\pi t)$, and ${\hat{x}}(t)$ is the decoded approximation to *x*(*t*).

The RMSE for the decoded spectrogram is computed with:

16 $$\begin{aligned} RMSE = \sqrt{\int _0^F \int _0^T \left( {\hat{p}}(f,t)-p(f,t)\right) ^2\,dtdf} \end{aligned}$$

where *p*(*f*, *t*) is the value of the song spectrogram at frequency *f* and time *t* over the range $f=0$ to $F=10$ kHz and from time $t=0$ time $T=0.88$ s (song duration). For each value of *N*, 10 trials (corresponding to different decoders) were used to obtain the average value of the RMSE for that fixed value of *N*. The linear fits to the RMSE were constructed on the loglog scale with the MATLAB *polyfit* function, for sufficiently large networks ($N\ge 2^{10}$).

### Measuring the stereotypy in the zebra finch networks

To estimate the variability from song-bout to song-bout, we computed the integrated variance, ${\overline{\sigma }}_{SB}$:

17 $$\begin{aligned} {\overline{s}}^2 = \int _F \int _0^T \sigma _{SB}^2(f,t)\,dtdf \end{aligned}$$

where

18 $$\begin{aligned} \sigma _{SB}^2(f,t)= & {} \sum _{k=1}^m \left( p_k(f,t) - \langle p_k(f,t) \rangle \right) ^2 \end{aligned}$$

19 $$\begin{aligned} \langle p_k(f,t) \rangle= & {} \frac{1}{m} \sum _{k=1}^m p_k(f,t) \end{aligned}$$

where ${\hat{p}}_k(f,t)$ denotes the *k*th trial’s decoded spectrogram for $k=1,2,\ldots 10$.

### Time discretization

To simulate the filtered spike-trains and decoded signal over a time interval [0, *T*] we discretize time into *M* evenly distributed points $\left( T_1,\dots ,T_M \right)$ with step size $\Delta t$, which we took as $\Delta t = 10^{-5}$ ms, and $T_k = (k\Delta )$ and $M = T/\Delta t$ . We then have a corresponding discretized filtered spike-train vectors $\left( {\varvec{r}}^1, \dots ,{\varvec{r}}^M \right)$ which are defined by:

20 $$\begin{aligned} r_j^k = \sum _{t_{ji} < T_k} K\left( T_k - t_{ji} \right) \end{aligned}$$

where *K* is the filtering function and $r_j^k$ is the $j^{th}$ neuron’s filtered spike-train at time step *k* and $t_{ji}$ is the *i*th spike fired by the *j*th neuron. We then generate the decoded output for each time step $T_k$ by the vector multiplication ${\hat{x}}=( \varvec{\phi }^ x)^T{\varvec{r}}^k$. We can improve the efficiency of this calculation for the exponential filter

21 $$\begin{aligned} K(t) = \exp \left( -\frac{t}{\tau _s} \right) \end{aligned}$$

using the exponentiation identity $e^{x+y}=e^xe^y$. If for neuron *j*, no spikes occur in the time interval $[T_{k-1},T_k]$ then we have the update

22 $$\begin{aligned} r_j^k = r_{j}^{k-1}\exp \left( - \frac{\Delta t}{\tau _s} \right) \end{aligned}$$

and if a spike does occur in the interval $[T_{k-1},T_k]$ at time $t_{ji}$ then we have the update

23 $$\begin{aligned} r_j^k = \exp \left( - \frac{T_k-t_{ji}}{\tau _s} \right) + r_{j}^{k-1}\exp \left( - \frac{\Delta t}{\tau _s} \right) \end{aligned}$$

Thus at each time step we do not need to compute the sum over all previous spikes and can instead perform a single update. For Fig. 6, the spike interpolation scheme in between time steps in Eq. 23 was not used, and a basic time step of $\Delta _t = 10^{-5}$ was utilized.

### Training/Testing trials

For Figs. 1, 2, 4 and 5, Supplementary Figs. S3–S4, and (6 songbird) a single trial of spikes was used to train the optimal linear decoders $\phi x$, while for Fig. 3, a total of 10 trials were used to train the optimal linear decoders. The test RMSE for Fig. 3 used 10 trials for 10 different random network seeds (spike trains). The test RMSE for Figs. 1, 2, 4 and 5, and Supplementary Figs. S3–S4, 10 different network seeds were used with 1 trial to estimate the test RMSE. For all simulated networks with the exception of the songbird networks (6), the trials used for training/testing were appended consecutively in a large matrix for $\varvec{r}(t)$. To avoid the initial transients for $\varvec{r}(t)=0$, an initial buffer trial of spikes was added. This buffer trial would be used to initialize the filtered spike trains $\varvec{r}(t)$ but would not be used to train the optimal linear decoders.

### Supplementary Information

Supplementary Information.

## Data Availability

A hyperlink to the songbird spectrogram can be found on the modelDB^46^ link above, with the raw recording from^47^. The raw recording was processed as in^14^.

## Code availability

The relevant code to generate the results of this paper can be found on modelDB^46^, under Accession no. 2015409 (direct hyperlink https://modeldb.science/2015409). The read-only access code for referees is ro1331. The accompanying code on modelDB will be released to the public after publication of this manuscript.
