# Supplementary material for: The impact of spike timing precision and spike emission reliability on decoding accuracy
Source: Sci Rep. 2024 May 8;14:10536. doi: 10.1038/s41598-024-58524-7 (PMC11078995; doi:10.1038/s41598-024-58524-7)
Supplement: Supplementary file 1 — Supplementary Information. [file 41598_2024_58524_MOESM1_ESM.pdf]

Supplementary Materials

Supplementary Material S1: Error Scaling with Precise Spike Times

Here, we will show for a general class of spike trains with precisely timed spikes generated by N neurons, the following result holds:

$$RMSE = \|\hat{x}(t) - x(t)\| = \sqrt{\int_0^T (\hat{x}(t) - x(t))^2 \, dt} \quad \propto \quad N^{-1} \tag{1}$$

where $\hat{x}(t)$ is the neurally decoded approximation to $x(t)$, given by the following:

$$\hat{x}(t) = \sum_{i=1}^{N} \phi_i^x r_i(t) \, dt. \tag{2}$$

where ϕ_i^x is the optimal decoder for $x(t)$.

The term $r_i(t)$ is the filtered sequence of spike times for neuron i:

$$r_i(t) = \sum_{t_{ij} < t} K(t - t_{ij})$$

where $K(t)$ is some filtering function. For the sets of simulations considered here, the filtering function $K(t)$ is the single exponential synaptic filter

$$K(t) = \exp(-t/\tau_s) \tag{3}$$

where $\tau_s = 10$ ms.

The derivation of (1) will be broken into two steps. In the first step, we will prove (1) for the case where each neuron fires a single spike, and the spikes are uniformly spread over the interval $[0, T]$. In the second step, we will prove that the same result holds for more general spike rasters by using linear transformations to determine when a general spike raster can be transformed into the evenly distributed one. The following derivation follows largely from classical approaches from functional analysis, the theory of function approximation, and the Simple Function Approximation Theorem [1, 2]. The $\frac{1}{N}$ in the RMSE scaling is indeed the "unavoidable discretization error" stated by [3], and numerically demonstrated in [3] (Figure 1a of [3], the regular-rate code).

Step 1: Evenly Distributed Spikes

Suppose the spike train fired by the N neurons is evenly distributed over an interval $[0, T]$, reminiscent of the HVC_{RA} projection neurons [4]. Thus, N neurons fire at successive $\Delta = \frac{T}{N}$ where T is the total duration of the signal to be approximated, $x(t)$. Each neuron fires at times $t_j = t_{j-1} + \Delta$ Suppose further more that we decode each spike with a box filter:

$$r_j(t) = \begin{cases} 1 & t \in [t_j, t_j + \Delta] \\ 0 & \text{otherwise.} \end{cases}$$

In order to approximate the signal $x(t)$, on an interval $[0, T]$, we need to determine what the decoders $\phi_j^x(t)$ for each neuron j. The decoders are easily resolved as the intervals $[t_{j-1}, t_j]$ are non-overlapping, and with box-filtering, the decoded spikes $r_j(t)$ are orthogonal. This immediately yields the following decomposition of the approximant:

$$\begin{aligned} \hat{x}(t) &= \sum_{j=1}^{N} \phi_j^x r_j(t) \\ \phi_j^x &= \frac{1}{\Delta} \int_{t_j}^{t_j + \Delta} x(s) \, ds \end{aligned}$$

The formula for the decoders is easily derived when one considers the orthogonality of the spike train. Note that if we determine the order of error in Δ, then with $\Delta = T/N$ we determine how the error scales with the network size. The squared error is thus:

$$\begin{aligned} E^2 &= \int_0^T \left(\hat{x}(s) - x(s)\right)^2 \, ds \\ &= \sum_j \int_{t_j}^{t_j+\Delta} \left(\phi_j^x - x(s)\right)^2 \, ds \end{aligned} \tag{4}$$

Now, if we can analytically determine or bound the integral

$$A_j = \int_{t_j}^{t_j+\Delta} (\phi_j^x - x(s))^2 \, ds$$

then we can bound the error in equation (4). First, note that by the mean-value theorem for integrals, on the interval $[t_j, t_j + \Delta]$ there exists a $c_j \in [t_j, t_j + \Delta]$

$$A_j = \int_{t_j}^{t_j+\Delta} (\phi_j^x - x(s))^2 \, ds = (\phi_j^x - x(c_j))^2 \Delta$$

ϕ_i^x is the mean value $x(t)$ over $[t_j, t_j + \Delta]$, by definition, thus by the intermediate value theorem, there must exist some c_j^* such that $x(c_j^*) = \phi_j^x$, then we have

$$A_i = (x(c_j^*) - x(c_j))^2 \Delta$$

We use the mean-value theorem for derivatives on the smaller interval $[c_j^*, c_j]$. We can assume without loss of generality that $c_j^* < c_j$, as the opposite case is entirely identical. If we assume that $x(t)$ is differentiable, then the mean-value theorem tells us there exists some $d \in [c_j^*, c_j]$ such that:

$$\frac{x(c_j^*) - x(c_j)}{c_j^* - c_j} = x'(d_j)$$

and thus:

$$A_j = x'(d_j)^2 (c_j^* - c_j)^2 \, \Delta \leq (x'(d_j))^2 \Delta^3$$

where the inequality comes from the fact that the interval $[c_j^*, c_j]$ lies within $[t_j, t_j + \Delta]$ and thus $c_j - c_j^* \leq \Delta$.

This yields the following:

$$\begin{aligned} E^2 = \sum_{j=1}^N A_j &\leq \Delta^3 \sum_{i=1}^N x'(d_i)^2 \\ &\leq \Delta^3 N \max_{d \in [0,T]} x'(d)^2 \\ &= \frac{T^3}{N^2} \max_{d \in [0,T]} x'(d)^2 \end{aligned}$$

Thus, we have the following:

$$\|\hat{x}(t) - x(t)\| \leq \frac{(\sqrt{T})^3}{N} \max_{d \in [0,T]} |x'(d)| \tag{5}$$

Result (5) implies that for uniformly distributed, precisely timed spikes, the RMSE in approximating a function is inversely proportional to the network size. Doubling the network size halves error, unlike in a conventional rate-code, where the network must quadruple in size to halve the RMSE. We also note that the condition that $x(t)$ must be differentiable can be relaxed to $x(t)$ being Lipschitz as then:

$$\begin{aligned} |x(c_j^*) - x(c_j)| &\leq K|c_j^* - c_j| \\ \rightarrow A_j &\leq K^2\Delta^3 \end{aligned}$$

with the rest of the derivation following along similar lines.

Step 2: Use Step 1 To Derive Result for More General Spike Trains

More generally, we will assume that the spikes are not uniformly distributed, however the time intervals defined above, $[t_j, t_j + \Delta]$, $j = 1, 2, \ldots N$ remain, and we still consider a network of N neurons. Thus, the following matrix emerges:

$$\hat{r}_{ij} = \begin{cases} 1 & t^* \in [t_i, t_i + \Delta] \\ 0 & t^* \notin [t_i, t_i + \Delta] \end{cases}, \quad i, j = 1, 2, \ldots N$$

where element (i, j) of $\hat{r}_{ij}$ is 1 if neuron j fires a spike (t^*) in the ith time interval, and 0 otherwise. Finally, we will assume that $\hat{\boldsymbol{r}}$ is an invertible matrix, or equivalently, the rank of $\hat{\boldsymbol{r}}$ is N. Note that if we consider the matrix generated for the uniformly distributed spiking case above $(\boldsymbol{r})$, then $\boldsymbol{r} = \boldsymbol{I}_N$ where $\boldsymbol{I}_N$ is the $N \times N$ identity matrix.

Then, if the matrix $\hat{\boldsymbol{r}}$ is invertible, consider the decoder defined by:

$$\boldsymbol{\psi}^{\boldsymbol{x}} = (\hat{\boldsymbol{r}}^{-1})^T \boldsymbol{\phi}^{\boldsymbol{x}}$$

where $\boldsymbol{\phi}^{\boldsymbol{x}}$ is the same decoder as in the uniformly distributed spiking case considered above. Applying $\boldsymbol{\psi}^{\boldsymbol{x}}$ to $\hat{\boldsymbol{r}}$ yields

$$(\boldsymbol{\psi}^{\boldsymbol{x}})^T \hat{\boldsymbol{r}} = \boldsymbol{\phi}_x^T (\hat{\boldsymbol{r}}^{-1} \hat{\boldsymbol{r}}) = \boldsymbol{\phi}_x^T \boldsymbol{I}_N = \phi_x^T \boldsymbol{r}$$

which restores the uniformly distributed spiking approximation in Step 1.

Now, consider the optimal linear decoder for the spike train $\hat{\boldsymbol{r}}$, as given by $\tilde{\boldsymbol{\psi}^{\boldsymbol{x}}}$. Then, we have the following:

$$\begin{aligned} \sqrt{\int_0^T \left(\sum_{j=1}^N \tilde{\psi^x}_j \hat{r}_j(t) - x(t) \right)^2 dt} &\leq \sqrt{\int_0^T \left(\sum_{j=1}^N \psi^x{}_j \hat{r}_j(t) - x(t) \right)^2 dt} \\ &= \sqrt{\int_0^T \left(\sum_{j=1}^N \phi_j^x r_j(t) - x(t) \right)^2 dt} \\ &\leq \frac{(\sqrt{T})^3}{N} \max_{d \in [0,T]} |x'(d)| \end{aligned} \qquad (6)$$

Thus, result (6) shows that any invertible spike-train is bounded by an $O(N^{-1})$ error. This is in principle most randomly generated spike trains for sufficiently large N as these matrices are highly likely to be full-rank (see for example [5, 6].

As a final comment, we note that this result has an implication for trained spiking neural networks with linear decoder-construction based approaches [7–16]. In particular, if the timing of spikes are stabilized to be precisely reproducible between training and testing phases, then $O(N^{-1})$ is the expected result. This, however, is not a necessary criterion, as under error-correcting spike-based codes (such as [3, 17, 18]), $O(N^{-1})$ convergence can still be achieved without precisely repeating spikes.

RMSE Scaling with Spike Failure

Given a signal $x(t)$, the optimal linear decoded approximation $\hat{x}(t)$ is defined as the lionear approximation to $x(t)$ using a basis of N evenly distributed spikes with box-filters on $[0, T]$ as:

$$
\begin{aligned}
\hat{x}(t) &= \sum_{j=1}^{N} \phi_j^x r_j(t)] \\
\phi_j^x &= \frac{1}{\Delta} \int_{t_j}^{t_j+\Delta} x(s)\, ds \qquad (7) \\
r_j(t) &= \begin{cases} 1 & t \in [t_j, t_j + \Delta] \\ 0 & \text{otherwise.} \end{cases} \qquad (8)
\end{aligned}
$$

It is assumed that each spike fails with probability p_F, which can be modelled by a Bernolli random variable $B_j \sim B(1, 1 - p_F)$ for each spike. Then, the estimated signal under spike failure is

$$
\tilde{x}(t) = \sum_{j=1}^{N} \phi_j^x B_j r_j(t).
$$

Next, we compute the root mean squared error (RMSE) for a given failure rate. The mean squared error (MSE) is given by:

$$
\begin{aligned}
MSE &= \mathbb{E}_B \left[\int_0^T \left(\tilde{x}(t) - x(t)\right)^2 dt \right] \\
&= \mathbb{E}_B \left[\int_0^T \left(\tilde{x}(t) - \hat{x}(t) + \hat{x}(t) - x(t)\right)^2 dt \right] \\
&= \mathbb{E}_B \left[\int_0^T \left(\tilde{x}(t) - \hat{x}(t)\right)^2 dt \right] + 2 \int_0^T \mathbb{E}_B \left[\tilde{x}(t) - \hat{x}(t)\right] \left(\hat{x}(t) - x(t)\right) dt + \int_0^T \left(\hat{x}(t) - x(t)\right)^2 dt \\
&= Var\left(\tilde{x}\right) + Cross(\tilde{x}, \hat{x}) + \left(Bias(\hat{x})\right)^2
\end{aligned}
$$

We have already established that the bias scales like N^{-1} (See S1). The variance is given by:

$$
\begin{aligned}
Var\left(\tilde{x}\right) &= \mathbb{E}_B \left[\sum_{j=1}^{N} \int_{t_j}^{t_j+\Delta} \left(B_j \phi_j^x - \phi_j^x\right)^2 dt \right] \\
&= \sum_{j=1}^{N} \int_{t_j}^{t_j+\Delta} \mathbb{E}_B \left[(B_j - 1)^2 \right] (\phi_j^x)^2\, dt \\
&= \sum_{j=1}^{N} \int_{t_j}^{t_j+\Delta} (p_F) \left(\phi_j^x\right)^2 dt \\
&= p_F \Delta \sum_{j=1}^{N} \left(\phi_j^x\right)^2
\end{aligned}
$$

As each decoder ϕ_j^x is the mean value of the function $x(s)$ over the interval $[t_j, t_j + \Delta]$, then by the mean value theorem for integrals, for each interval $[t_j, t_j + \Delta]$ there exists an $s_j \in [t_j, t_j + \Delta]$ where $\phi_j^x = x(s_j)$. Further, the variance can be bounded by assuming that $x(s)$ has a maximum, M, on $[0, T]$ (a consequence of the extreme value theorem). Thus:

$$
Var(\tilde{x}) = \Delta p_F \sum_{j=1}^{N} (x(s_j))^2 \leq \Delta p_F M^2 N = T M^2 p_F
$$

For the cross term:

$$\begin{aligned}
Cross(\tilde{x}, \hat{x}) &= 2\sum_{j=1}^{N}\int_{t_j}^{t_j+\Delta} \mathbb{E}_B\left[B_j\phi_j^x - \phi_j^x\right]\left(\phi_j^x - x(t)\right)\,dt \\
&= 2\sum_{j=1}^{N}\phi_j^x\left(\Delta\phi_j^x - \int_{t_j}^{t_j+\Delta} x(t)\,dt\right) \\
&= 2\sum_{j=1}^{N} -p_F\phi_j^x\left(\Delta\phi_j^x - \Delta\phi_j^x\right) \\
&= 0
\end{aligned}$$

Thus, we have

$$RMSE^2 = MSE = \left(Bias(\hat{x})\right)^2 + Var(\tilde{x}) \leq \left(Bias(\hat{x})\right)^2 + TM^2 p_F$$

As the bias scales like N^{-1}, in order for the RMSE to also scale like N^{-1}, it is sufficient for the probability of spike failure, p_F to scale like N^{-2} as $N \to \infty$. Note that in this derivation, there is no assumed spike-redundancy. If a single spike fails, there are no other spikes in the interval $[t_j, t_j + \Delta]$ to serve in place of the failed spike.

RMSE Scaling for Differentiable Basis with Spike Jitter

Given a signal $x(t)$ on an interval $[0, T]$ and a basis of N differentiable functions $r_j(t)$, constructed from filtering spike times, the approximant $\hat{x}(t)$ is defined as:

$$\begin{aligned}
\hat{x}(t) &= \sum_{j=1}^{N}\phi_j^x r_j(t) \\
\phi^x &= \left(\int_0^T \mathbf{r}(t)\mathbf{r}(t)^T\,dt\right)^{-1}\int_0^T \mathbf{r}(t)x(t)\,dt
\end{aligned}$$

We then consider the effect of random jitter applied to each of the basis elements on the mean squared error (MSE) scaling. In this derivation, it is assumed that the spikes fired by a single neuron are not jittered independently. Each random jitter δ_j in the jth basis element is assumed to be normally distributed with probability density $\mathcal{N}(0, \sigma^2)$. Then, define the jittered basis $\tilde{r}_j(t)$ and decoded signal $\tilde{x}(t)$ to be:

$$\begin{aligned}
\tilde{r}_j(t) &= r_j(t + \delta_j) \\
\tilde{x}(t) &= \sum_{j=1}^{N}\phi_j^x\tilde{r}_j\left(t\right)
\end{aligned}$$

The mean squared error (MSE) can be decomposed into the bias squared, variance, and a cross-term:

$$\begin{aligned}
MSE &= \mathbb{E}_\delta\left[\int_0^T \left(\tilde{x}(t) - x(t)\right)^2\,dt\right] \\
&= \mathbb{E}_\delta\left[\int_0^T \left(\tilde{x}(t) - \hat{x}(t) + \hat{x}(t) - x(t)\right)^2\,dt\right] \\
&= \mathbb{E}_\delta\left[\int_0^T \left(\tilde{x}(t) - \hat{x}(t)\right)^2\,dt\right] + 2\mathbb{E}_\delta\left[\int_0^T \left(\tilde{x}(t) - \hat{x}(t)\right)\left(\hat{x}(t) - x(t)\right)\,dt\right] \\
&\quad + \int_0^T \left(\hat{x}(t) - x(t)\right)^2\,dt \\
&= Var(\tilde{x}) + Cross(\hat{x}, \tilde{x}) + \left(Bias(\hat{x})\right)^2
\end{aligned}$$

We have established that the bias scales like N^{-1} (See S1). Next, we investigate the scaling of the variance and cross-term. First, we consider the effect of a single perturbation δ_j on a single basis element. If we $r_j(t)$ is differentiable and $|r_j'(t)|$ has a maximum M, then we can apply a Taylor expansion to o $r_j(t+\delta_j)$ centered at t:

$$\begin{aligned}|r_j\left(t+\delta_j\right)-r_j\left(t\right)| =& \left|r_j(t)+\delta_j r_j'(t)+O(\delta_j^2)-r_j(t)\right| \\ \leq& M|\delta_j|\end{aligned}$$

where M is the maximum of the derivative of the filtered spikes, $r_j(t)$. We can then consider the impact of N perturbations $\delta_1,\ldots,\delta_N$ to the variance:

$$\begin{aligned}Var\left(\tilde{x}\right) &= \mathbb{E}_\delta\left[\int_0^T\left|\sum_{j=1}^N \phi_j^x\left(r_j\left(t+\delta_j\right)-r_j(t)\right)\right|^2 dt\right] \\ &\leq \mathbb{E}_\delta\left[\int_0^T\left(\sum_{j=1}^N \left|\phi_j^x\right|\left|r_j\left(t+\delta_j\right)-r_j(t)\right|\right)^2 dt\right] \\ &\leq \mathbb{E}_\delta\left[\int_0^T\left(\sum_{j=1}^N \left|\phi_j^x\right|\left|\delta_j\right|M\right)^2 dt\right] \\ &= \mathbb{E}_\delta\left[\int_0^T\sum_{j=1}^N\sum_{i=1}^N \left|\phi_j^x\right|\left|\phi_i^x\right|\left|\delta_j\right|\left|\delta_i\right|M^2\,dt\right] \\ &= TM^2\left[\sum_{j=1}^N\left(\phi_j^x\right)^2\mathbb{E}_\delta\left[\delta_1^2\right]+2\sum_{j=1}^N\sum_{i=1}^{j-1}\left|\phi_j^x\right|\left|\phi_i^x\right|\mathbb{E}_\delta\left[\left|\delta_1\right|\right]^2\right]\end{aligned}$$

We can now use the fact that $\mathbb{E}_\delta\left[\delta^2\right]=\sigma^2$ and $\mathbb{E}_\delta\left[|\delta|\right]=\sigma\sqrt{\frac{2}{\pi}}\leq\sigma$ to get:

$$\begin{aligned}Var(\tilde{x}) &\leq TM^2\sigma^2\left[\sum_{j=1}^N\left(\phi_j^x\right)^2+2\sum_{j=1}^N\sum_{i=1}^{j-1}\left|\phi_j^x\right|\left|\phi_i^x\right|\right] \\ &= TM^2\sigma^2\sum_{j=1}^N\sum_{i=1}^N\left|\phi_j^x\right|\left|\phi_i^x\right|\end{aligned}$$

For the cross-term we can apply Hölder's inequality to get

$$\begin{aligned}Cross(\hat{x},\tilde{x}) &\leq 2\left|\mathbb{E}_\delta\left[\int_0^T\left(\tilde{x}(t)-\hat{x}(t)\right)\left(\hat{x}(t)-x(t)\right)dt\right]\right| \\ &\leq 2\mathbb{E}_\delta\left[\int_0^T\left|\tilde{x}(t)-\hat{x}(t)\right|\left|\hat{x}(t)-x(t)\right|dt\right] \\ &\leq 2\mathbb{E}_\delta\left[\left(\int_0^T\left|\tilde{x}(t)-\hat{x}(t)\right|^2dt\right)^{1/2}\right]\left(\int_0^T\left|\hat{x}(t)-x(t)\right|^2dt\right)^{1/2} \\ &= 2Bias\left(\hat{x}\right)\sqrt{Var\left(\tilde{x}\right)}\end{aligned}$$

Thus, overall we get

$$MSE \leq TM^2\sigma^2\sum_{j=1}^N\sum_{i=1}^N|\phi_j^x||\phi_i^x|+2Bias(\hat{x})M\sigma\sqrt{T\sum_{j=1}^N\sum_{i=1}^N\left|\phi_j^x\right|\left|\phi_i^x\right|+Bias(\hat{x})^2} \tag{9}$$

Thus, if the quantity

$$\sum_{j=1}^N\sum_{i=1}^N|\phi_j^x||\phi_i^x| = O(1) \tag{10}$$

and if $\sigma \propto N^{-1}$, then the RMSE will scale like N^{-1} as all terms in the bias variance decomposition will individually scale like N^{-1}. Note that equation (10) need not be satisfied by every possible spike train generated and supervisor $x(t)$ generated. For example, if two filtered spike trains are linearly dependent for two neurons, neuron m and n, then the optimal decoder is not uniquely specified. For example, consider

$$r_n(t) = r_m(t)$$

$$\begin{aligned}
\hat{x}(t) &= \sum_{j \neq n,m}^{N} \phi_j r_j(t) + \phi_n r_n(t) + \phi_m r_m(t) \\
\hat{x}(t) &= \sum_{j \neq n,m}^{N} \phi_j r_j(t) + (\phi_n + \Psi) r_n(t) + (\phi_m - \Psi) r_m(t) \\
&= \sum_{j \neq n,m}^{N} \phi_j r_j(t) + \tilde{\phi_n} r_n(t) + \tilde{\phi_m} r_m(t) \\
&= \hat{x}(t)
\end{aligned}$$

where Ψ is any real number. Thus, the decoders in this scenario need not be bounded. As a result, ϕ_m and ϕ_n can become arbitrarily large. In general, condition (10) will likely depend on the basis elements/spike trains generated.

RMSE Scaling for Non-Smooth Functions

Consider the step function $H(t)$, where:

$$H(t) = \begin{cases} 1 & t > t^* \\ 0 & t \leq t^* \end{cases}$$

for some $t^* \in (0,1)$. Now, consider the uniformly distributed, box-filtered basis set defined by:

$$r_i(t) = \begin{cases} 1 & t \in [t_i, t_{i+1}] \\ 0 & \text{otherwise} \end{cases}$$

With the linear decoder $\boldsymbol{\phi}^H$, where $\hat{H}(t) = \boldsymbol{\phi}^T \boldsymbol{r}(t)$ This decoder is determined by minimizing the following:

$$\begin{aligned}
MSE &= \int_0^1 \left(\hat{H}(t) - H(t)\right)^2 dt \\
&= \int_0^1 \left(\boldsymbol{\phi}^T \boldsymbol{r}(t) - H(t)\right)^2 dt \\
&= \int_0^{t_i} \left(\boldsymbol{\phi}^T \boldsymbol{r}(t) - H(t)\right)^2 dt + \int_{t_{i+1}}^1 \left(\boldsymbol{\phi}^T \boldsymbol{r}(t) - H(t)\right)^2 dt + \int_{t_i}^{t_{i+1}} (\boldsymbol{\phi}_i - H(t))^2 \, dt
\end{aligned}$$

where t_i is the unique interval start-point that satisfies $t_i \leq t^* < t_{i+1}$. The first two terms are minimized and equal to 0 by setting $\phi_j = 0$ for $t_j < t_i$ and $\phi_j = 1$ for $t_j \geq t_{i+1}$. Then, the loss is:

$$\begin{aligned}
MSE &= \int_{t_i}^{t_{i+1}} (\boldsymbol{\phi}_i - H(t))^2 \, dt \\
&= \int_{t_i}^{t^*} (\boldsymbol{\phi}_i)^2 \, dt + \int_{t^*}^{t_{i+1}} (\boldsymbol{\phi}_i - 1)^2 \, dt \\
&= \phi_i^2 (t^* - t_i) + (\phi_i - 1)^2 (t_{i+1} - t^*)
\end{aligned}$$

The optimum ϕ_i is given by:

$$\frac{\partial MSE}{\partial \phi_i} = 2\phi_i(t^* - t_i) + 2(\phi_i - 1)(t_{i+1} - t^*) = 0 \tag{11}$$
$$\phi_i^* = \frac{(t_{i+1} - t^*)}{\Delta}$$

now since $\phi^2 \leq \phi$, $(\phi - 1)^2 \leq 1 - \phi$, $t^* - t_i \leq \Delta$ and $t_{i+1} - t^* \leq \Delta$ we get:

$$L(\phi_i^*) = \phi_i^2 \,(t^* - t_i) + (1 - \phi_i)^2 \,(t_{i+1} - t^*) \leq \phi_i\Delta + (1 - \phi_i)\Delta = \Delta = \frac{1}{N}$$

Thus, the RMSE for a heaviside function is expected to scale like $\sqrt{N}^{-1}$ with box-filters is expected to scale like $\sqrt{N}^{-1}$.

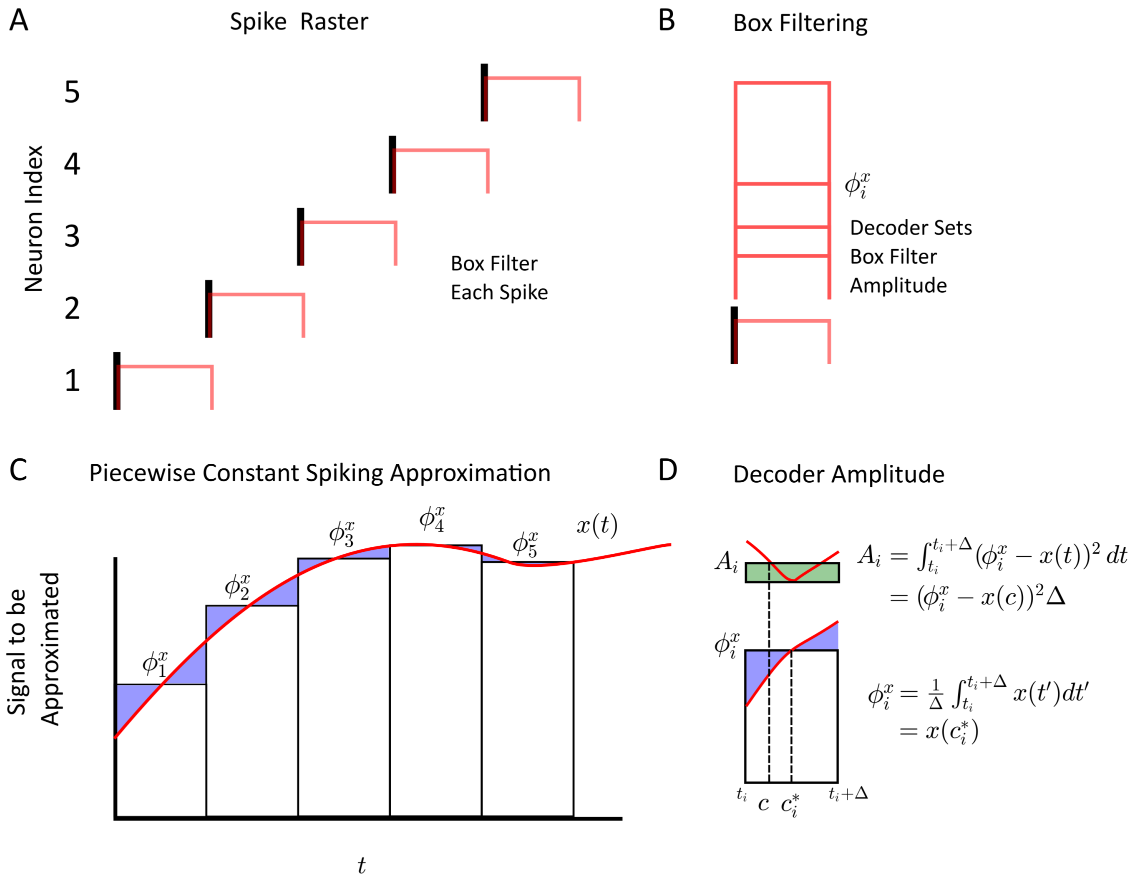

Supplementary Figure S1: **(A)** The proof in the supplementary material for $O(N^{-1})$ scaling relies on a chain of individual spikes which are box filtered to transform the inherently discrete spikes into a signal that covers a bin of time. **(B)** The optimal decoder for box-filtered, orthogonal spikes becomes the amplitude of each individual bin value. **(C)** The bins approximate continuous functions by setting the decoder value to the mean of the function value over the discrete-time bin a single spike covers. **(D)** Analytical determination of the decoder and application of the mean-value theorem for integrals.

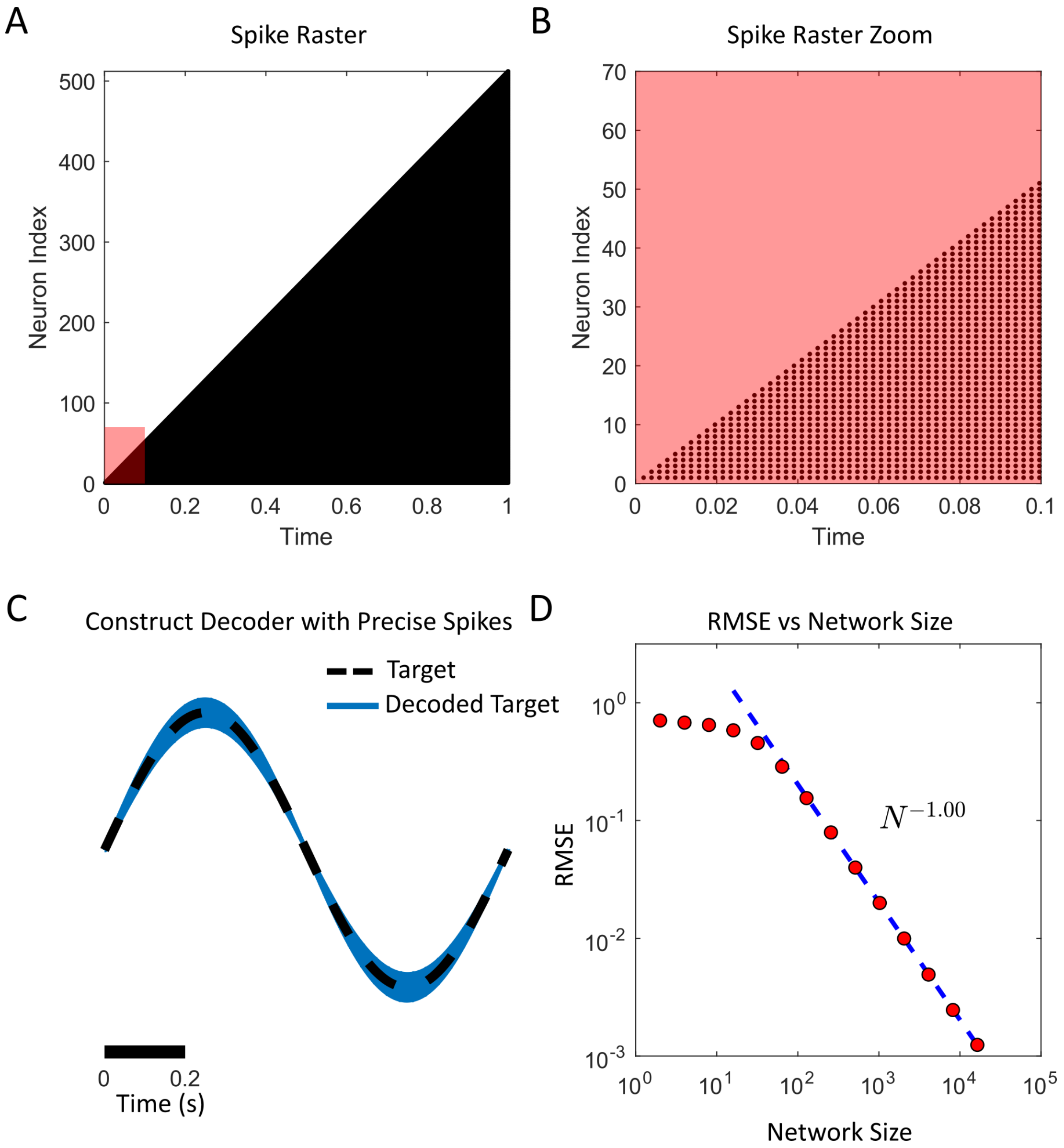

Supplementary Figure S2: **(A)** The spike raster plot for $N = 512$ neurons over the interval $[0, 1]$ where neuron j first spikes precisely at $k/(N)$, for $k \geq j$. When the spikes are synthetically generated, any 2 neurons in the network will have filtered spikes that overlap with each other. **(B)** A zoom of the first 70 neurons spike raster from (A). **(C)** The linear decoder applied to (A) with $N = 512$ neurons. **(D)** The RMSE decreases linearly with the network size (RMSE $\propto N^{-1}$) when the spikes are generated as in (A) for larger N.

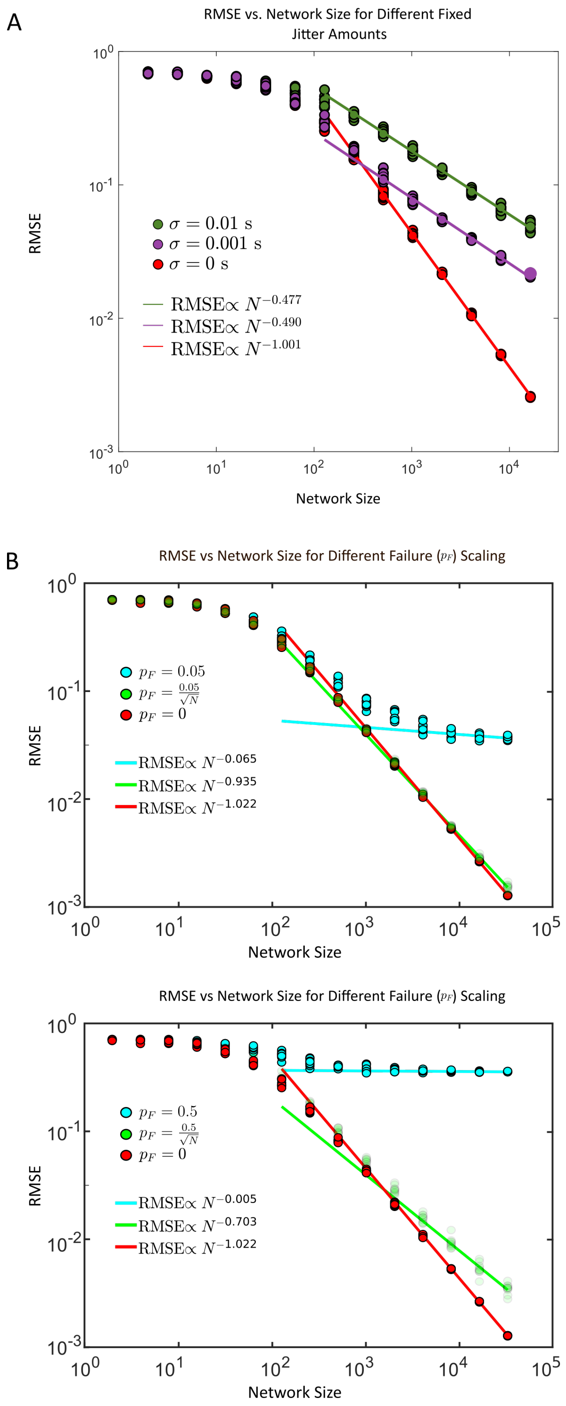

Supplementary Figure S3: **(A)** The root mean squared error as a function of the network size with spike jitter with a standard deviation of $\sigma = 0$s (red), and $\sigma = 1$ ms (purple), $\sigma = 10$ ms (green) for varying network sizes. As N increases, the RMSE decreases linearly for jitterless spikes (RMSE $\propto N^{-1.001}$) and sublinearly for jittered spikes with a fixed standard deviation of the jitter σ (RMSE $\propto N^{-0.477}$, $\sigma = 10$ ms, and RMSE $\propto N^{-0.490}$ for $\sigma = 1$ ms) **(B)** The RMSE for $p_F = 0.05$ and $p_F = 0.05/\sqrt{N}$ (top) and $p_F = 0.5$ and $p_F = 0.5/\sqrt{N}$ (bottom). The supervisor and additional parameter settings are identical as in Figure **??** (bottom).

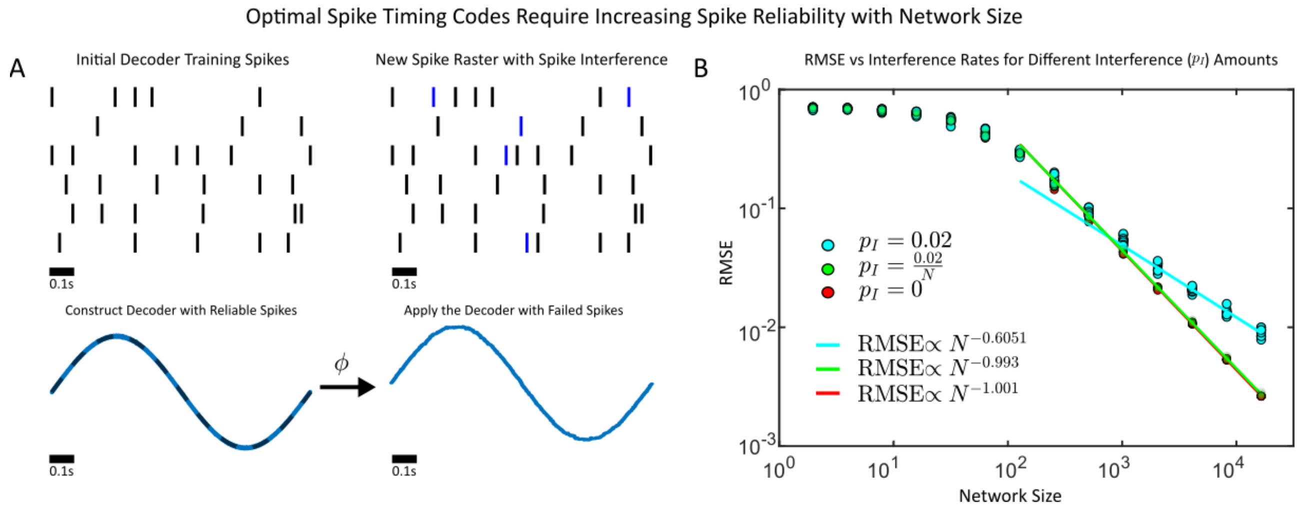

Supplementary Figure S4: **(A)** The impacts of spike interference on decoding accuracy. (Top) In spike interference, spikes are randomly activated at segments of time where they were not expected by the trained decoder. (Bottom) The initial spike train is used to construct a decoder. Spikes are then randomly added with the same decoder applied, and the resulting error is measured for varying network sizes. **(B)** The root mean squared error (RMSE) for the decoded signal (sinusoidal oscillator) for networks with fixed spike interference ($p_F = 0.02$, blue), no spike interference ($p_F = 0.00$, red) and increasingly reliable spikes $p_F = \frac{0.02}{\sqrt{N}}$ for larger networks, where p_F is the probability that a spike is randomly added (see Materials and Methods).

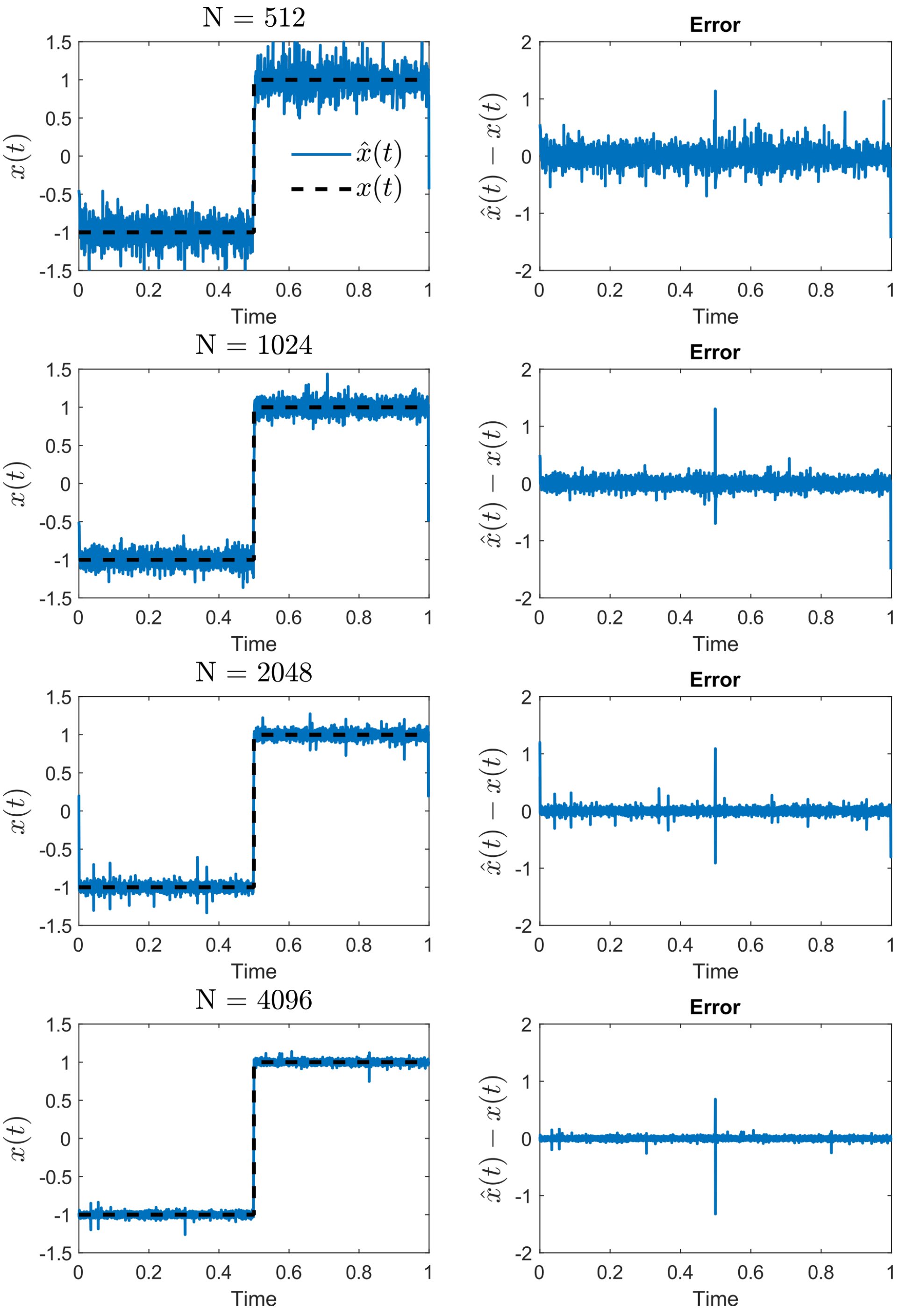

Supplementary Figure S5: The decoded sign function (left column). The dashed black line corresponds to $f(t) = sign(t - 0.5)$ while the decoded signal is in blue. The error $x(t) - \hat{x}(t)$ shows a distinct spike (right column) at the discontinuity.

References

[1] Kenneth R Davidson and Allan P Donsig. *Real analysis with real applications*. Prentice Hall, 2002.

[2] Walter Rudin. *Principles of mathematical analysis*. International series in pure and applied mathematics. McGraw-Hill, New York, 1976.

[3] Sophie Denève and Christian K Machens. Efficient codes and balanced networks. *Nature neuroscience*, 19(3):375, 2016.

[4] Richard HR Hahnloser, Alexay A Kozhevnikov, and Michale S Fee. An ultra-sparse code underliesthe generation of neural sequences in a songbird. *Nature*, 419(6902):65, 2002.

[5] Terence Tao and Van Vu. On the singularity probability of random bernoulli matrices. *Journal of the American Mathematical Society*, 20(3):603–628, 2007.

[6] Jean Bourgain, Van H Vu, and Philip Matchett Wood. On the singularity probability of discrete random matrices. *Journal of Functional Analysis*, 258(2):559–603, 2010.

[7] Wilten Nicola and Claudia Clopath. Supervised learning in spiking neural networks with force training. *Nature communications*, 8(1):1–15, 2017.

[8] Wilten Nicola and Claudia Clopath. A diversity of interneurons and hebbian plasticity facilitate rapid compressible learning in the hippocampus. *Nature Neuroscience*, 22(7):1168–1181, 2019.

[9] Larry F Abbott, Brian DePasquale, and Raoul-Martin Memmesheimer. Building functional networks of spiking model neurons. *Nature neuroscience*, 19(3):350–355, 2016.

[10] Brian DePasquale, Christopher J Cueva, Kanaka Rajan, G Sean Escola, and LF Abbott. full-force: A target-based method for training recurrent networks. *PloS one*, 13(2):e0191527, 2018.

[11] Dominik Thalmeier, Marvin Uhlmann, Hilbert J Kappen, and Raoul-Martin Memmesheimer. Learning universal computations with spikes. *PLoS computational biology*, 12(6):e1004895, 2016.

[12] Chris Eliasmith and Charles H Anderson. *Neural engineering: Computation, representation, and dynamics in neurobiological systems*. MIT press, 2003.

[13] Aditya Gilra and Wulfram Gerstner. Predicting non-linear dynamics by stable local learning in a recurrent spiking neural network. *Elife*, 6:e28295, 2017.

[14] Friedemann Zenke and Surya Ganguli. Superspike: Supervised learning in multilayer spiking neural networks. *Neural computation*, 30(6):1514–1541, 2018.

[15] Cynthia A Chestek, Aaron P Batista, Gopal Santhanam, M Yu Byron, Afsheen Afshar, John P Cunningham, Vikash Gilja, Stephen I Ryu, Mark M Churchland, and Krishna V Shenoy. Single-neuron stability during repeated reaching in macaque premotor cortex. *Journal of Neuroscience*, 27(40):10742–10750, 2007.

[16] Răzvan V Florian. The chronotron: A neuron that learns to fire temporally precise spike patterns. *PloS one*, 7(8):e40233, 2012.

[17] Martin Boerlin, Christian K Machens, and Sophie Denève. Predictive coding of dynamical variables in balanced spiking networks. *PLoS computational biology*, 9(11):e1003258, 2013.

[18] Michael A Schwemmer, Adrienne L Fairhall, Sophie Denéve, and Eric T Shea-Brown. Constructing precisely computing networks with biophysical spiking neurons. *Journal of Neuroscience*, 35(28):10112–10134, 2015.
